# The Intriguing Connection Between the Gut and Lung Microbiomes

**DOI:** 10.3390/pathogens13111005

**Published:** 2024-11-15

**Authors:** Magdalena Druszczynska, Beata Sadowska, Jakub Kulesza, Nikodem Gąsienica-Gliwa, Ewelina Kulesza, Marek Fol

**Affiliations:** 1 Department of Immunology and Infectious Biology, Faculty of Biology and Environmental Protection, Institute of Microbiology, Biotechnology and Immunology, University of Lodz, 90-237 Lodz, Poland; beata.sadowska@biol.uni.lodz.pl (B.S.); nikodem.gasienica.gliwa@edu.uni.lodz.pl (N.G.-G.); marek.fol@biol.uni.lodz.pl (M.F.); 2 Department of Internal Diseases and Clinical Pharmacology, Medical University of Lodz, 91-347 Lodz, Poland; jakub.kulesza@mp.pl; 3 Department of Rheumatology and Internal Diseases, Medical University of Lodz, 90-549 Lodz, Poland; ewelina.kulesza@mp.pl

**Keywords:** gut–lung axis, microbiome, respiratory diseases

## Abstract

Recent advances in microbiome research have uncovered a dynamic and complex connection between the gut and lungs, known as the gut–lung axis. This bidirectional communication network plays a critical role in modulating immune responses and maintaining respiratory health. Mediated by immune interactions, metabolic byproducts, and microbial communities in both organs, this axis demonstrates how gut-derived signals, such as metabolites and immune modulators, can reach the lung tissue via systemic circulation, influencing respiratory function and disease susceptibility. To explore the implications of this connection, we conducted a systematic review of studies published between 2001 and 2024 (with as much as nearly 60% covering the period 2020–2024), using keywords such as “gut–lung axis”, “microbiome”, “respiratory disease”, and “immune signaling”. Studies were selected based on their relevance to gut–lung communication mechanisms, the impact of dysbiosis, and the role of the gut microbiota in respiratory diseases. This review provides a comprehensive overview of the gut–lung microbiome axis, emphasizing its importance in regulating inflammatory and immune responses linked to respiratory health. Understanding this intricate pathway opens new avenues for microbiota-targeted therapeutic strategies, which could offer promising interventions for respiratory diseases like asthma, chronic obstructive pulmonary disease, and even infections. The insights gained through this research underscore the potential of the gut–lung axis as a novel target for preventative and therapeutic approaches in respiratory medicine, with implications for enhancing both gut and lung health.

## 1. Introduction

Traditionally, the intestine has been regarded primarily as a digestive organ, while the lungs are considered to be responsible for gas exchange and respiratory functions [1]. However, emerging evidence reveals a complex, bidirectional communication network between these systems, referred to as the gut–lung axis, which may significantly impact the function and health of both the gut and the respiratory system [2]. This connection suggests that the intestinal microbiota, which plays an essential role in metabolic processes and immune system regulation, might also influence respiratory health. The microbial communities in the gut coexist symbiotically with their host, utilizing available nutrients to perform critical tasks such as fermenting food components to produce metabolites, vitamins, and minerals. For instance, short-chain fatty acids (SCFAs) like lactic acid and butyric acid, produced through fermentation, serve as an energy source for large intestinal epithelial cells [3,4]. Gut bacteria also affect hepatic fat metabolism, indirectly influencing cholesterol and fatty acid transformation, while contributing to essential B vitamins and vitamin K synthesis [3]. Beyond these metabolic roles, the gut microbiota is integral to immune function, promoting the growth and maturation of immune cells through microbial sensing and providing local and systemic signals that help shape immune responses [5,6,7,8,9]. Through competition for habitat and nutrients and bacteriocin production, beneficial microbes prevent pathogenic bacteria from thriving and thereby support tissue homeostasis.

Given this context, a critical question arises: how might alterations in the gut microbiome influence respiratory health through the gut–lung axis? We hypothesize that dysbiosis, or an imbalance in gut microbiota, disrupts this axis and contributes to the development or exacerbation of respiratory diseases such as asthma, chronic obstructive pulmonary disease (COPD), and lung infections. This hypothesis is supported by emerging findings linking gut-derived metabolites and immune signaling molecules to inflammatory and immune responses in the lungs. In this review, we aim to explore the mechanisms underlying the gut–lung axis, with a focus on the microbiome’s role, immune mediators, and inflammatory pathways. We also delve into how dysbiosis in the gut microbiota may contribute to respiratory diseases and highlight potential therapeutic strategies targeting the gut microbiome to alleviate lung-related ailments and enhance respiratory health. To investigate the underlying mechanisms of this gut–lung connection and its health implications, we conducted a targeted review of relevant studies. Articles were selected from databases such as PubMed and Web of Science, covering the period from 2001 to 2024 (approximately 60% are from 2020 to 2024), using keywords including “gut–lung axis”, “microbiome”, “dysbiosis”, and “respiratory disease”. Inclusion criteria focused on studies that explored gut–lung communication mechanisms, immune and metabolic pathways, and the impact of gut dysbiosis on respiratory health. This approach allowed us to synthesize findings on how the gut microbiota influences immune responses in the lungs, focusing on both local and systemic effects.

## 2. Mechanisms of the Gut-Lung Axis

### 2.1. Immune System Interactions

**Figure 1:**
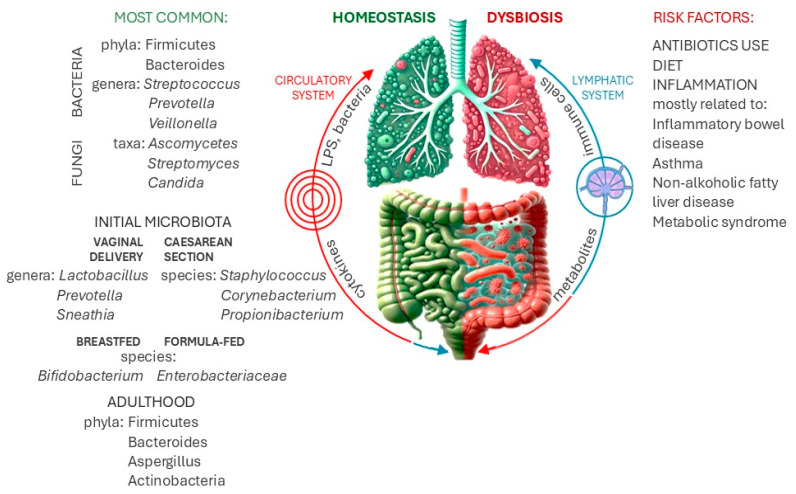
The gut–lung axis: interconnected immune regulation. The gastrointestinal tract is depicted with its diverse microbial communities producing metabolites that modulate lung immunity. In turn, respiratory infections or inflammation can disrupt the gut microbiota, leading to dysbiosis. Key components like immune cells (macrophages, dendritic cells) and inflammatory mediators (cytokines, chemokines) are messengers facilitating this bidirectional interaction. Imbalances in the gut microbiome may contribute to respiratory diseases such as asthma, inflammatory bowel disease, and lung infections.

In recent years, the gut microbiome, which has been the subject of extremely intensive research, appears to have been much better identified than the microbiome inhabiting the respiratory system, particularly the lungs. For decades, the lungs were considered a sterile area, and it was only with the development of modern research and diagnostic techniques, including computed tomography (CT) scans, polymerase chain reaction (PCR), and 16S rRNA sequencing, that this view gradually changed [10]. Research shows that there are interactions between both these structures, manifesting in their ability to influence the composition of the microbiota and immune response. When comparing both microbiomes, the most striking observation is the enormous disparity in the number of microorganisms constituting them. It is estimated that the microbial community of the gastrointestinal tract consists of more than 100 trillion microorganisms (the overwhelming majority being bacteria, but also fungi (mycobiota), viruses including phages (virobiota), archaea, protists, and helminths, with the most intensively colonized part being the colon, which has an estimated density of 10^11^ to 10^12^ bacteria per milliliter) [11,12]. Meanwhile, the lung microbiome represents a much smaller community, estimated at 10 to 100 bacteria per 1000 human cells [13]. The microbiota composition is influenced by factors that appear in the earliest stages of life, such as the type of delivery, birth gestational date, mode of birth, method of feeding, and the timing of weaning, as well as factors that become more important with age, including body mass index (BMI), frequency of exercise, lifestyle and cultural and dietary habits, and medications taken [14]. All of this means that, while the microbiota composition is largely stable, it is subject to inter-individual and intra-individual differences throughout a person’s life, impacting communication within the gut–lung axis. This communication occurs in both health and disease states. Multiple gut disorders, such as inflammatory bowel disease (IBD), ulcerative colitis, and Crohn’s disease, have been associated with respiratory conditions including asthma, chronic obstructive pulmonary disease (COPD), and cystic fibrosis [15,16]. One of the most intensively studied levels of communication within the gut–lung axis (GLA) is dysbiosis, whether related to the gut or lungs, which is influenced by factors such as antibiotic use, stress, diet, and metabolic diseases [17]. The balance in the lungs and gut is determined by the integrity of the epithelial membrane barrier, the presence of a stable and diverse microbiota, the efficiency of macrophages (which play a crucial role in the state of lung tissue), and the regulatory properties of T lymphocytes present in the intestinal lamina propria. Inflammatory conditions caused by dysbiosis lead to the apoptosis of intestinal epithelial cells, disruption of the tight junctions of the epithelium, and ultimately increased intestinal permeability. In these conditions, there is increased exposure not only to microbial metabolites but also to a wide range of cytokines and chemokines such as, e.g., tumor necrosis factor α (TNF-α), transforming growth factor β (TGF-β), interleukin (IL)-1β, IL-5, IL-6, IL-8, IL-13, IL-17, IL-18, IL-33, C-C motif chemokine ligand (CCL) 2, 3, 4, 7, 20, C-X-C motif chemokine ligand (CXCL) 5, 8, 10, RANTES (regulated on activation, normally T-cell-expressed and secreted, also known as CCL5), produced by immune cells within the epithelial layer of the gut mucosa These products are distributed through both the circulatory system and the lymphatic vessels of the mesentery and mediastinum. The result is an influx into the intestines, particularly of neutrophils and T lymphocytes, forming lymphoid aggregates, which are a source of immune cells infiltrating other organs, including the lungs. Regarding T lymphocytes, attention is drawn to the CCR9 (C-C motif chemokine receptor) and α4β7 molecules, which may enable this translocation to the lungs. Humoral factors delivered by the lymphatic vessels also reach the lungs, resulting in the activation of alveolar macrophages, promoting an inflammatory environment that ultimately leads to damage to the alveolar barrier. Microbial metabolites can also be distributed via a similar route, affecting the functioning of both lung epithelial cells and immune cells [17]. It has been demonstrated in a mouse model that, through the damaged gut epithelial barrier, LPS (lipopolysaccharide) can reach the lungs, leading to an LPS-induced acute lung injury (ALI) by regulating the TLR (Toll-like receptor) 4/NF-κB pathway and increasing the production of IL-1β, IL-6, and TNF-α. Fecal microbiota transplantation (FMT) has shown a beneficial effect, most likely by restoring a balanced gut microbiota composition, including an increase in the number of beneficial/commensal bacteria that produce SCFAs (short-chain fatty acids), which potentially inhibit the activation of the TLR 4/NF-κB pathway and reduce the production of pro-inflammatory factors and oxidative stress [18]. In the context of the perturbation of the gut microbiome, an extremely interesting topic is the effect of SCFAs, specifically butyrate, on the development and severity of allergic asthma [19]. In humans, the primary source of butyrate is directly through diet and the fermentation processes of commensal bacteria: *Ruminococcaceae*, *Lachnospiraceaes*, *Erysipelotrichaeceae*, and *Clostridiaceae* (Figure 1). Hence, the highest concentration of butyrate is in the large intestinal lumen (~100 mmol/kg chyme) and intestinal tissue (~25 mmol/kg tissue), while in the bloodstream it reaches ~1–10 μM. It has been shown that butyrate may exhibit beneficial properties in asthmatic conditions by acting on various immunological mechanisms: inhibiting dendritic cell activation and their migration to local lymph nodes where the stimulation of naive CD4+ T lymphocytes occurs, resulting in polarization toward T helper (Th) 2 and limiting plasma cell differentiation and class-switching to IgE antibodies, as well as reducing mast cell degranulation as a result of IgE binding, promoting a regulatory T lymphocyte (Tregs) phenotype by converting Th9 cells to FoxP3+ Tregs, and finally exerting a wide range of effects on eosinophils by inhibiting their adhesion to endothelial cells, thus limiting their migration (especially in response to CCL24), directly promoting their apoptosis, and reducing IL-5 and IL-13 expression by ILC2 (innate lymphoid cells), which also negatively affects eosinophils. These processes were discussed in detail by Yip et al. [19]. Other microorganisms present in the gut microbiota, namely segmented filamentous bacteria (SFB), may stimulate the development of pathological processes in the lungs [20]. Discovered for the first time in the mid-1960s in laboratory animals, SFB, whose characteristic biological hallmark appears to be host specificity, have proven to be key players in shaping immune processes, whether by stimulating the production of chemokines and antimicrobial components, inducing gut lymphoid tissue, and causing a strong increase in fecal IgA, or through their potent triggering of Th17 cell differentiation [21]. It has been shown in a mouse model that SFB-associated lung pathology requires activation of Th17 cells. SFB-induced Th17 cells from the gut are preferentially recruited to the lungs instead of the spleen because of the strong expression of the Th17 chemoattractant, CCL20, in the lungs. Moreover, in peripheral tissues, SFB selectively proliferate dual Th17 cells, which have TCRs (T-cell receptors) capable of recognizing both SFB epitopes and epitopes of the host, increasing the risk of autoimmunity [20]. It turns out that, within the gut–lung axis, interactions can also occur in the lung-to-gut direction, not just gut-to-lung [10]. Based on a mouse model where animals were administered LPS intratracheally, it was shown that, in addition to the increased presence of neutrophils in BALF (bronchoalveolar lavage fluid), there were changes in the intestines, manifesting as an increased number of CD45+ cells in the ileal mucosa, a rise in goblet cell expression, and enhanced expression of EpCAM (epithelial cell adhesion molecule). This suggests that lung inflammation may influence intestinal inflammation and the integrity of the intestinal barrier. At the same time, LPS did not cause changes at the level of the enteric nervous system expressed as alterations in the expression of glial markers such as GFAP (glial fibrillary acidic protein), S100β, or the number of neuronal markers Hu+ and nNOS (neuronal nitric oxide synthase) in the myenteric plexus [22]. Goossens et al., in a mouse model of LPS-induced systemic inflammatory response syndrome (SIRS), in which one of the most common and earliest affected organs are the lungs, showed that there was 15.49% and 24.09% variation in the microbiota of the jejunum and ileum, respectively, while there was no effect of LPS provocation on the microbial community structure of the colon [23]. In the jejunum, a major alteration was noticed regarding the phylum *Actinobacteriota*, which decreased from 23.44% in the unchallenged mice to 9.78% after the LPS. In the case of the phyla *Bacteroidota*, *Firmicutes*, and *Proteobacteria*, an increase was reported together with a decrease in *Verrucomicrobiota*. In the ileal microbiome, the highest shift was observed regarding Proteobacteria from 0.44% in the unchallenged mice to 6.37% and 45.7% at 4 h and 8 h after the LPS challenge, which indicates dysbiosis [23]. Furthermore, some respiratory viral infections can affect the gut microbiome’s composition by inducing the production of IFNs (interferons) in the lung, as was detailed by Lane et al. [24].

### 2.2. Microbial Crosstalk

Complex gut microbial community and its metabolites condition homeostasis in the gastrointestinal tract, assisting in digestion and energy acquisition, the absorption of nutrients, macro-, and microelements, and vitamin production, modulating the tightness of the intestinal barrier, and stimulating the development of intestinal lymphoid tissue. The gut microbiota also supports the proper immune response of the whole organism, which requires local (intestinal) tolerance to external antigens and, at the same time, activation of peripheral/systemic defense mechanisms [25,26,27]. Thus, these microorganisms not only modulate gastrointestinal functioning and immunity but also impact distal organs like the lungs, affecting the lung microbiota, homeostasis, and the immune state of the respiratory system [28,29,30,31,32]. The microbiota of the gut and lungs are engaged in a bi-directional crosstalk that influences the host’s overall health. The gastrointestinal and respiratory tracts connect directly through the mouth and pharynx, maintain indirect contact through lymph nodes and the bloodstream, and also have a similar organization and physiology of mucus membranes arising from the same origin [26]. All these aspects allow crosstalk between the gut and lung microbiota. Microbial interactions are based primarily on cross-feeding processes and products such as SCFAs, or may involve the activation of host immune cells. For example, *Ruminococcus bromii* and *Eubacterium rectale* are the main starch degraders in the gut microbiota, leading to the release of oligo- and monosaccharides that can be used by themselves or with other bacteria for growth and SCFA production [33]. SCFAs (mainly propionate, acetate, and butyrate) maintain the proper functioning of the intestinal barrier, regulate glucose and lipid metabolism, alleviate oxidative stress and inflammation, and are described as one the main modulators of the gut and lungs’ immunity [26,27,30,32,34]. Their effect on immune cells that play a role in maintaining homeostasis in the GLA has been briefly described above. Interestingly, the local production and accumulation of SCFAs in the lung mucosa after reaching the bloodstream are limited. Thus, the gut microbiota is the main source of SCFAs influencing immune cells in lamina propria and mesenteric lymph nodes. Then, these cells arrive in the respiratory system through circulation [26]. The effect of SCFAs on hematopoietic precursor production in the bone marrow to keep homeostasis in the lungs and alleviate potential airway inflammation has also been described. Propionate produced in mice during a fiber-rich diet stimulated macrophages and dendritic cell progenitors, which later were able to trigger phagocytosis, but not to trigger Th2-mediated allergic airway inflammation [29,35]. Microbial crosstalk as part of the gut–lung axis involving the activation of host immune cells has mainly been demonstrated in mouse models. It was found, for instance, that segmented filamentous bacteria within the gut microbiota protect against acute methicillin-resistant *Staphylococcus aureus* (MRSA) pneumonia in C57BL/6 mice by promoting pulmonary type 17 immunity. A lack of these bacteria in the gut led to higher MRSA burdens in the lungs, lung inflammation, and a higher rate of mice mortality [36]. McAleer et al., on a C57BL/6 mice model, demonstrated that segmented filamentous bacteria also regulate lung immune responses to *Aspergillus fumigatus* pulmonary infection through Th17 cells. Th17 cells primed by the gut microbiota reduced the production of inflammatory cytokines in lung tissue and protected mice from weight loss [37]. Negi et al. demonstrated that oral administration of *Lactobacillus plantarum* upregulated the expression of macrophage-inducible C-type lectin (Mincle) and major histocompatibility complex (MHC) II on lung dendritic cells, which was accompanied by an increase in the frequency of activated and effector memory CD4+ T cells leading to a reduction in lung *Mycobacterium tuberculosis* burden in infected mice with gut dysbiosis [38]. The global outbreak known as COVID-19 (coronavirus disease 2019) caused by severe acute respiratory syndrome coronavirus-2 (SARS-CoV-2) brought with it research on the link between gut microbiota status and the course of this viral infection. Several human studies reported that disturbances in the gut microbiota equilibrium resulted in a decrease in several beneficial commensals (e.g., *Faecalibacterium*, *Eubacterium*, *Roseburia*, *Lactobacillus*) and an increase in opportunistic pathogens (e.g., bacteria from the order Enterobacterales, *Enterococcus*) in COVID-19 patients, which positively correlated with SARS-CoV-2 load, aggravated inflammation, and COVID-19 severity. Knowledge in this area can be found in many recently published review articles [39,40,41,42]. Thus, changes in the gut microbiota, including diet-associated modifications or post-antibiotic dysbiosis, can not only lead to many gastrointestinal dysfunctions (e.g., nutritional disorders, inflammatory bowel diseases, necrotizing enterocolitis, bacterial infections), but also alterations in the lung microbiota and immune response in the respiratory tract, which in turn can exacerbate respiratory chronic disease, acute infections, and worsen the condition of the host.

#### Gut and Lung Microbiota Composition

Both the gastrointestinal and respiratory tracts host multi-species communities of microorganisms, although the gut microbiota is more abundant and diverse than the lung microbiota. The gut microbiota is the richest and the most complex microbial community in the human body and comprises trillions of microorganisms, including bacteria, archaea, fungi, viruses, and protozoa [26,43,44]. Until recently, it was thought that the formation of the gut microbiota begins right after birth. However, the presence of microorganisms in the uterus, amniotic fluid, placental and fetal membranes suggests the microbial colonization of the fetus during pregnancy [45]. Based on both culture methods and 16S rRNA microarrays, predominantly *Bacillus* sp., *Staphylococcus* sp., *Streptococcus mitis*, *Escherichia fergusonii* and *Lactobacillus* sp. from the phylum *Firmicutes* were detected in meconium [46,47,48]. A high individual variability was also found depending on the condition of the mother. Maternal diabetes status enriched the presence of bacteria from the phylum *Bacteroidetes* and the genus *Parabacteroides* in samples of meconium [45]. After birth, the composition of the gut microbiota changes, which is highly dependent on how the baby is fed. In breastfed infants, higher amounts of *Bifidobacterium*, *Lactobacillus*, *Staphylococcus*, and *Streptococcus* species have been noted, while in infants who are formula-fed *Bacteroides* sp., *Clostridium* sp., and *Proteobacteria* predominate [28,49]. The core composition of the adult gut microbiota is fairly stable in healthy organisms throughout their lifetime. However, the numerical ratios of the different groups change depending on the diet and lifestyle. Generally, 90% of the adult gut microbiota belongs to the phyla *Firmicutes* and *Bacteroidetes.* Among the *Firmicutes* are genera such as *Streptococcus*, *Eubacterium*, *Ruminococcus*, *Lactobacillus*, *Enterococcus*, *Veillonella*, and *Clostridium*, while *Bacteroides* and *Prevotella* predominate among the *Bacteroidetes*, as well as *Bifidobacterium* belonging to the phylum *Actinobacteria* [26,44,50]. Diet can significantly change the gut microbiota composition, thus affecting homeostasis in this complex ecosystem. This action also includes the disruption of the synthesis of bioactive metabolites such as SCFAs, which, being important immunomodulators, exert effects on both epithelial and immune cells in the gut and lungs [33,43,51]. A Western diet rich in animal proteins, carbohydrates, and fats decreases the prevalence of *Bifidobacterium*, *Eubacterium*, and *Roseburia* species, increasing *Bacteroides* sp. and *Clostridium* sp. A Mediterranean diet rich in plant proteins, beneficial unsaturated fatty acids, fiber, and polyphenols increases *Lactobacillus*, *Bifidobacterium*, and *Prevotella* species, reducing pathogenic *Bacteroides fragilis* and *Clostridium* sp. [28,43,44,51]. The diverse gut community composition, cross-feeding processes, and diet determine the final SCFA types and levels. Bacteria such as *R. bromii* or *E. rectale* are producers of acetate and formate or butyrate, respectively [33].

The lung microbiota is much smaller than the gut microbiota, develops from the microorganisms inhaled with air or translocated from the digestive tract, and is mainly located in the upper respiratory tract. The composition of the lung microbiota depends on the microbiological cleanliness of the air, contact with other microbial carriers (people or animals), the host’s ability to control the local proliferation of the microorganisms and their elimination, and the host’s immune status [52,53]. Though less dense than the gut, the lung microbiota includes a variety of microorganisms that can influence respiratory health, help maintain immune homeostasis, and prevent infections. The predominant microbial phyla in the respiratory tract include *Firmicutes* and *Actinobacteria* in the nasal cavity, *Firmicutes*, *Proteobacteria*, and *Bacteroidetes* in the oropharynx, and *Bacteroidetes* and *Firmicutes* in the lungs [54,55]. The microbiota in the upper respiratory tract have a high biomass and includes mainly *Streptococcus*, *Staphylococcus*, *Haemophilus*, *Fusobacterium*, *Moraxella*, *Neisseria*, *Corynebacterium*, *Alloprevotella*, *Dolosigranulum*, while the relatively low-biomass microbiota the in lower respiratory tract consist of *Prevotella* (up to 50%), *Streptococcus*, *Veillonella*, *Haemophilus*, *Fusobacterium*, and *Neisseria* [31,54,56,57].

### 2.3. Metabolic and Neural Pathways

Different metabolic pathways play critical roles in the interactions between the digestive and respiratory systems. These mechanisms involve a complex network of biochemical processes, including exchanging metabolites, hormones, and signaling molecules between the gut and the lungs. One of the essential metabolic products is SCFAs, which are produced by the gut microbiota’s metabolism of indigestible nutrients (e.g., dietary fiber) [4]. Most SCFAs, such as acetate, propionate, and butyrate, are consumed by colonocytes for energy or used by epithelial cells in the gut to shape local immunity. Unmetabolized SCFAs are later redistributed from the liver by circulation to peripheral tissues [58]. SCFAs have been shown to have anti-inflammatory and immunomodulatory effects, which may impact lung health and respiratory function. Among others, SCFAs affect immune cell development in the bone marrow, inhibit dendritic cell activity, influence regulatory T cell differentiation, and reduce neutrophil recruitment to inflammatory sites [59,60,61]. Another important pathway in the gut–lung communication network is the production of neurotransmitters and neuropeptides by the enteric nervous system in the gut. These signaling molecules can influence the activity of the respiratory system through the vagus nerve, which connects the gut and the lungs, and are responsible for regulating various physiological processes, such as immune responses, inflammation, and the coordination of the gut and the lungs’ functions [62]. Additionally, metabolites such as bile acids, lipids, and amino acids produced in the gut can also impact lung function and immune responses. Bile acids have been shown to have antimicrobial properties and can modulate the function of immune cells in the lungs [63]. The gut microbiota can interact with the lungs through soluble microbial components and bacterial metabolites called pathogen-associated molecular patterns (PAMPs) that enter the bloodstream and can affect both metabolic and neurological pathways [8]. PAMPs are recognized by host cells that express pattern recognition receptors (PRRs), such as nucleotide-binding and oligomerization domain (NOD) receptors (NLRs) or Toll-like receptors (TLRs) [64]. TLRs and NLRs are widely expressed on various immunological and epithelial cells that, after activation, trigger a signaling cascade to deal with invasive pathogens and/or heal injured tissue. Excessive receptor activation disturbs immunological homeostasis and causes pro-inflammatory mediators to be continuously produced, which raises the risk of autoimmune and inflammatory disorders [65].

## 3. Implications for Health and Disease

### 3.1. Asthma and Allergies

A long-term and healthy (normal) intestinal microbiota is formed around the age of 2 years of the child and depends on factors such as the method of birth—natural or by cesarean section—breastfeeding, and the use of antibiotics. Studies suggest that one-year-old children are most likely to develop asthma through dysbiosis [66]. Early-onset asthma has been observed in children aged 3–6 months [67]. The fecal microbiome of these children has been studied, and a reduction in the level of the metabolite 3-ketosphinganine was noted. It has been proven that this decrease is due to a reduced amount of *Bacteroides* in the gut microbiome being producers of this metabolite [67]. Factors that could affect the early onset of asthma were selected by modifying the gut microbiota in children aged 3–6 months, 1 year, 3 years. These factors include perinatal antibiotics used, method of delivery (natural or cesarean section), breastfeeding, and contact with pets. What is beyond doubt is that breastfeeding affects the gut environment outside the microbiome (including its biochemical properties) and slightly affects the risk of asthma. It is not possible to directly determine whether the method of delivery adversely affects the occurrence of asthma in the child; however, a reduced number of *Bacteroides* has been observed in children born by cesarean section, which may increase the risk of early-onset asthma through dysbiosis. Perinatal antibiotics and dog ownership have not been found to affect fecal bacteria in children [67]. There is a potential way in which microbial therapy can prevent the development of asthma, through the use of probiotics. These conclusions were made based on studies on mouse models, which were treated with four bacterial taxa *Lachnospira*, *Veillonella*, *Faecalibacterium*, and *Rothia*, which made it possible to alleviate inflammation of the airways in mice. These bacterial genera were used because studies in infants at risk of asthma who showed gut bacterial dysbiosis were noted to have a reduced abundance of these four bacterial taxa [68].

During infancy, the gut microbiota shapes future food allergies in children. To prove this, children up to 8 years of age were tested for the occurrence of a food allergy to milk [69]. The experiment included children who had acquired resistance to milk and children who were allergic to milk. It was confirmed that, in children, the enrichment of the gut microbiome by the *Firmicutes* cluster and the *Clostridia* class at the age of 3 to 6 months allowed the allergy to disappear at the age of 8 years (Table 1). In contrast, children whose milk allergy did not resolve had increased levels of *Bacteroides* and *Enterobacter* [69]. Factors affecting the gut microbiome in children (such as perinatal factors, cesarean birth, breastfeeding, and atopic skin disease in parents) were screened for symptoms of allergic eczema, food allergy, allergic rhinitis, and asthma in children who were monitored up to 13 years of age. The clearest results concerned the occurrence of allergic rhinitis in children, the occurrence of which is mainly influenced by whether both parents have atopic skin disease. However, focusing on changes in the gut microbiome of children, it was found that the greatest impact on allergic rhinitis occurrence is a reduced number of *Bifidobacterium*. Factors affecting the reduction in their number include antibiotic use up to 6 months of age, birth by cesarean section, and to the least extent, atopic skin disease in both parents [70]. *Escherichia*/*Shigella* bacteria had an inhibitory effect on allergic rhinitis occurrence in children, the growth of which is stimulated by the occurrence of an atopic skin disease in both parents and the use of antibiotics until the child is 6 months old. On the other hand, factors that have a positive effect on the number of *Bifidobacterium* are breastfeeding and the use of probiotics, which also reduce the number of *Escherichia*/*Shigella*. Based on the results obtained, treatment with a probiotic product consisting of a mixture of *Lactobacillus*, *Bifidobacterium*, and *Propionibacterium* spp. complemented by oligosaccharides was proposed to reduce the risk of allergic rhinitis in children [70].

### 3.2. Chronic Obstructive Pulmonary Disease (COPD)

COPD, a chronic lung disease with a diverse phenotype and various underlying mechanisms [71,72,73,74] has been linked to the composition of the gut microbiota and its alterations. Wang et al. reported that patients with COPD had distinct gut microbiota profiles compared to healthy individuals, suggesting a role of the gut microbiome in the disease’s pathogenesis and progression [75]. According to the latest data, COPD is responsible for as many as 3 million deaths worldwide, with an average mortality rate of 42/100,000. The incidence is expected to increase in the coming decades, especially in highly developed countries [76]. Lai et al., on a mouse model of cigarette smoking-induced COPD (CS-COPD), demonstrated the possibility of affecting the gut–pulmonary axis, whether through the use of antibiotics that modify the composition of the natural intestinal microbiota or the transplantation of the microbiota [77]. Using a wide range of antibiotics, it was shown that, in contrast to neomycin (NEO) and metronidazole (MET), oral administration of vancomycin (VAN), ampicillin (AMP), or their combination (MIX) significantly alleviated the lesions accompanying CS-COPD which was perfectly evident in a histopathological image of lung tissue. Thus, the composition of the gut microbiota is able to effectively influence the disease state of COPD patients. Treatment with VAN and AMP or MIX, in contrast to treatment with the other antibiotics, was accompanied by a decrease in IL-1β and TNF-α production by F4/80+CD11b+ macrophages and IL-17A in CD4+NKp46+ Th17 cells and an increase in IL-10 production by Th17 cells. Importantly, the effect achieved by using antibiotic therapy appeared to be transferable, as shown by experiments with the transfer of fecal microbiota. It was shown that the fecal microbiota of mice treated with MIX, AMP, or VAN, but not NEO, was able to reverse COPD traits in CS-recipients. Using the 16S microbiota ribosomal sequencing technique, it was shown that in CS-COPD mice there was a reduction in the abundance of microorganisms such as *Erysipelotrichaceae*, *Bacteroidales* and *Ruminococcaceae*, and an increase in *Lachnospiriaceae*, compared to the control mice (Table 2). Interesting data on the relationship between COPD and gut microbiota composition were provided by a large-scale shotgun metagenomics study involving sequencing and taxonomic profiling stool samples from several thousand participants with machine learning to develop prediction models at different taxonomic levels separately [78]. The samples were collected from January to March 2002 by the Finnish Institute for Health and Welfare and then sequenced at the University of California San Diego in 2017. The study allowed for the classification of 151 phyla, 338 classes, 925 orders, 2254 families, 7906 genera, and 24,705 species according to the Genome Taxonomy Database (GTDB); however, two phyla, *Firmicutes* and *Bacteroidetes*, were the most common. They were dominated by the *Clostridia* and *Bacteroides* classes, respectively, with the most abundant genera including *Faecalibacterium*, *Agathobacter*, *Bacteroides*, and *Prevotella* [78]. Analysis using various statistical tools (baseline alpha-diversity measures, principal-component analysis of the centered log-ratio (CLR)-transformed abundances) showed that the potential association between the occurrence of COPD and the gut microbiome should be attributed to specific microbial taxa rather than to the microbial community as a whole. It was found that the increased abundance of genera such as *Faecalicatena*, *Oscillibacter*, *Lawsonibacter*, *Flavonifractor*, and *Streptomyces* and reduced abundances of *Lachnospira*, *Eubacterium*, and *Coprococcus* were associated with incident COPD. Furthermore, the gut microbiome score had a relatively high predictive capacity with a C-index of 0.817, which was greater than those of other risk factors (sex, age, body mass index (BMI), smoking) [78]. There is a need for further research into the impact of the gut microbiome on the severity and progression of COPD, which may yield clinically meaningful findings, including the establishment of microbiome biomarkers that can improve COPD risk profiling.

### 3.3. Tuberculosis (TB)

Studies in mice and humans show that the lung microbiome plays a role in resistance to *Mycobacterium tuberculosis* (*Mtb*), the cause of tuberculosis [79,80,81]. *Mtb*-infected individuals have a reduced microbiota diversity compared to healthy controls and often show an enrichment of *Streptococcus* and *Pseudomonas*. In addition, the presence of *Pseudomonas* is associated with an increased risk of treatment failure. *Mtb* infection dysregulates the immune system, leading to an altered gut microbiome [82]. A study comparing the gut microbiome of adult TB patients with healthy controls found a reduction in *Firmicutes*, *Proteobacteria*, and *Verrucomicrobia* types, while there was an increase in *Actinobacteria*, *Bacteroidetes* and *Fusobacteria* [83]. In another study, analyzing patients with new and recurrent TB, there was a reduction in *Bacteroidetes*, *Prevotella*, and *Lachnospira* genera and an enrichment in *Actinobacteria* and *Proteobacteria* [84]. Finally, a reduction in *Actinobacteria* and *Firmicutes* of the genera *Bifidobacterium*, *Dorea*, *Faecalibacterium*, *Ruminococcus*, and *F. prausnitzii* and an increase in *Bacteroides*, *Proteobacteria*, *Enterococcus*, and *Prevotella* were observed in a group of sick children (Table 3). *Lactobacillus* supplementation can restore anti-tumor immunity depending on the dendritic cells in the lungs. Oral treatment with *Akkermansia muciniphila* or *A. muciniphila*-dependent palmitoleic acid strongly inhibited tuberculosis infection via the epigenetic suppression of TNF in *Mtb*-infected mice [82,85]. The bacterial diversity in the guts of TB patients is altered, which may correlate with disease progression. The severity of *Mtb* infection seems to be linked with the gut microbiota [1]. Anti-tuberculosis therapy includes antibiotics, such as rifampicin, which target also bacteria other than mycobacteria. Prolonged anti-tuberculosis treatment has been shown to alter the gut microbiota of patients and the resulting dysbiotic state persists after the cessation of therapy. This suggests that prolonged anti-tuberculosis treatment up to at least six months may make patients more susceptible to other disorders and infections [86].

### 3.4. COVID-19

Intestinal dysbiosis in patients infected with SARS-CoV-2 has been associated with the progression and severity of COVID-19 disease and is characterized by reduced numbers of anti-inflammatory bacteria, such as *Bifidobacterium* and *Faecalibacterium*, and reduced numbers of butyrate producers, such as several genera from the *Ruminococcaceae* and *Lachnospiraceae* families (Table 4). In addition, an excessive growth of inflammation-related opportunistic bacterial pathogens such as *Streptococcus*, *Rothia* and *Actinomyces* has been reported [82,87,88]. The gut microbiota may also regulate angiotensin-converting enzyme 2 (ACE2) receptor expression in the colon. This may help to explain the increased susceptibility to disease and gastrointestinal symptoms in people with gut dysbiosis, such as the elderly, immunocompromised patients, and patients with other comorbidities [82,89,90]. A protective role of *Bacteroides* spp. as an important member of the intestinal microbiota against COVID-19 infection by reducing ACE2 expression and limiting SARS-CoV-2 entry is indicated. Increased levels of ACE2 expression may promote viral entry, whereas its reduction inhibits the ACE2-Ang1-7-Mas pathway and further protects against lung injury during SARS-CoV-2 infection [88,91,92]. In a retrospective study of intensive care unit patients with SARS-CoV-2 pneumonia, treatment with a probiotic mixture containing *Lactobacillus*, *Bifidobacterium* and *Streptococcus* species showed a positive association with reduced mortality compared to standard care [82,93,94,95]. A study in critically ill patients showed that administration of synbiotics reduced the number of days in the intensive care unit and days when patients required mechanical ventilation [89]. Oral administration of the probiotic *Lactobacillus helveticus* positively modulated the immune system and had an immunoprotective effect on mucosal immunity by increasing the number of IgA-secreting cells in the gut and broncho-associated lymphoid tissue. Importantly, for COVID-19, it is now accepted that the local innate immune response, particularly secretory IgA, is the main defense mechanism in the early stages of infection [89]. In an open-label study of 55 hospitalized patients with COVID-19, a higher proportion of subjects (88% versus 63.3%) who received a synbiotic formula consisting of *Bifidobacterium* strains and prebiotics (SIM01) for 4 weeks achieved the resolution of clinical symptoms, an increase in IgG antibodies to SARS-CoV-2, and a reduction in blood pro-inflammatory markers such as IL-6, CCL2, M-CSF (macrophage colony-stimulating factor), TNF, and IL-1RA (interleukin-1 receptor antagonist) than subjects in the standard treatment arm. An increase in the abundance of commensal bacteria, such as *Bifidobacterium*, *Eubacterium*, and *Faecalibacterium* species, and a reduction in opportunistic pathogens, such as *E. coli* and *Bacteroides* species, was found in the gut microbiome of people who received SIM01. In addition, a cohort study of 200 people showed that regular consumption of probiotic yogurt in the year before COVID-19 was associated with a milder disease course [87]. COVID-19 patients also had reduced serum and fecal sphingolipid levels and altered gut microbial sphingolipid metabolism. Sphingolipids produced by *Bacteroides* may increase exogenous sphingolipids and thus enhance the differentiation of regulatory T cells, as observed in vitro or in vivo, which may inhibit coronavirus replication. On the other hand, an analysis of the nasopharyngeal microbiomes of patients suffering from acute respiratory illness did not show differences in composition or diversity when comparing patients with confirmed COVID-19 with those who were negative. This suggests that SARS-CoV-2 infection does not significantly alter the microbiome compared to the healthy state. Therefore, the diversity of the respiratory microbiota in patients with COVID-19 remains a matter of debate [82].

### 3.5. Other Viral and Bacterial Infections

#### 3.5.1. Influenza Virus

Experimental studies indicate that the commensal microbiota regulates the production of virus-specific CD4+ and CD8+ T cells and antibody responses following influenza virus infection, while a depletion of gut bacteria due to antibiotic treatment increases susceptibility to influenza infection [89]. Takeda et al. observed that oral administration of 10 strains of lactic acid bacteria isolated from traditional Mongolian dairy products to influenza-infected mice alleviated the symptoms of infection through immunomodulatory effects [96]. Feeding *Bifidobacterium longum* MM-2 isolates to influenza-infected mice reduced inflammatory responses in the lower respiratory tract and mortality by activating NK cells in the lungs and spleen and increasing cytokine expression in the lungs (Table 5). Intranasal or oral administration of *Lactobacillus plantarum* DK119 protected against a lethal dose of influenza A virus by modulating dendritic cell and macrophage activity and increasing IL-12 and IFN-γ levels in bronchoalveolar lavage fluid [91]. *L. paracasei* has been reported to increase dendritic cell recruitment in lung tissue after influenza A virus (IAV) infection. *L. rhamnosus* M21 reduces inflammatory damage in the lungs of IAV-infected mice and increases IFN-γ and IL-2 levels in lung lysates. Combined treatment with the probiotic *L. mucosae* 1025 and *B. breve* CCFM1026 increased stool butyrate levels in avian influenza (AI) virus-infected mice and attenuated inflammatory infiltration into lung tissue [97]. Studies showed that certain strains of *Lactobacilli* such as *L. casei* stimulated lung NK cells [98]. The probiotics *L. rhamnosus* and *L. brevis* have been found to be associated with a reduction in the incidence of influenza infections [95]. However, in a study of 523 children aged 2–6 years, daily consumption of a milk drink containing *L. rhamnosus* GG for 28 weeks did not reduce the incidence of various respiratory viral infections, including influenza virus, or respiratory symptoms [89].

#### 3.5.2. Respiratory Syncytial Virus (RSV)

In a mouse model of RSV, a change in microbiome diversity with an increase in Bacteroidetes and a decrease in *Firmicutes* was described [82,99]. This increase in Bacteroidetes was mainly due to an increase in the *Bacteroidaceae* while the decreased abundance of Firmicutes was associated with a weakening of the *Lachnospiraceae* and *Lactobacillaceae* families (Table 6). Probiotics, such as *Lactobacillus*, *Bifidobacterium*, *Enterococcus*, or *Lactococcus*, administered before RSV infection, resulted in symptom relief and improved survival [82]. *L. mucosae* inhibited RSV replication and reduced the proportion of inflammatory cells in the blood, such as granulocytes and monocytes. Colonization of the gut by one common bacterial species, namely SFB, reprogrammed AMs, conferring increased proliferation, complement production and phagocytosis, resulting in increased protection against RSV and SARS-CoV-2 infection [99].

#### 3.5.3. *Streptococcus pneumoniae*

Recent studies have illuminated the importance of this axis in the context of pulmonary infections, particularly those caused by pathogenic bacteria such as *Streptococcus pneumoniae* [27]. These Gram-positive bacteria are a major cause of pneumonia and other respiratory illnesses, and their impact on host immunity is profoundly influenced by the microbiota residing in the gut. A recent study exemplifies the interplay between the gut microbiota and susceptibility to *S. pneumoniae* infections [100]. In this experiment, wild-type mice were administered a broad spectrum of antibiotics (ampicillin, neomycin, metronidazole, and vancomycin) through their drinking water. This treatment effectively depleted their gut microbiota. Following this, the mice were intranasally infected with *S. pneumoniae.* The results were striking; mice lacking a gut microbiome exhibited accelerated mortality following the infection (Table 7). This finding underscores the protective role of the gut microbiota against severe outcomes in response to respiratory infections. To further elucidate the mechanisms involved, the cytokine levels were measured post-infection. Mice with depleted microbiota showed a significant increase in pro-inflammatory cytokines such as interleukin (IL)-1β, IL-6, and chemokine CXCL1. In contrast, there was a notable decrease in TNF-α and anti-inflammatory IL-10 levels within 6 h of the intranasal inoculation. Such an imbalance in the inflammatory response was exacerbated 48 h after infection, as the gut microbiota-depleted mice presented with heightened inflammation and substantial tissue damage [100]. These data emphasize the necessity of gut microbiota in mitigating inflammatory responses and indicates potential pathways through which microbiome modulation may offer therapeutic benefits. Interestingly, the ability of alveolar macrophages, crucial immune cells in the lungs, to phagocytose *S. pneumoniae* was adversely affected in mice deprived of their gut microbiota. The diminished phagocytic capacity of these immune cells in the absence of a healthy gut microbiome corroborates the idea that the gut microbiota enhances the functional activity of immune cells, particularly in the context of a lung infection. The protective role of the gut microbiota against *S. pneumoniae*-induced pneumonia has led to further investigations into therapeutic strategies. For instance, the intranasal administration of probiotics has shown promise in modulating the immune response. Treatment with probiotics resulted in increased local TNF-α and IFN-γ production, alongside a reduction in tissue damage [101]. These findings point towards the potential of probiotics as a promising adjunctive therapy in managing respiratory infections, potentially restoring immune balance and enhancing host defense mechanisms.

#### 3.5.4. *Pseudomonas aeruginosa*

*Pseudomonas aeruginosa* is a versatile and opportunistic pathogen that is particularly notorious for its role in respiratory infections, especially among immunocompromised individuals and those with underlying lung diseases such as cystic fibrosis. One emerging field of study about *P. aeruginosa’s* impact on health involves the gut–lung axis, a concept that explores the intricate relationships between the gut microbiota and lung health. In a study focused on this gut–lung connection, researchers investigated the potential protective effects of probiotics against *P. aeruginosa* infection [102]. Probiotics were first administered to mice via endotracheal instillation as a prophylaxis. Following this, an infection with *P. aeruginosa* was induced via the same route to observe the probiotics’ effects during an active infection. The study evaluated a mixture of three probiotics: *Lactobacillus fermentum*, *Lactobacillus paraesei*, and *Lactobacillus zeae* (Table 8). The results were promising: administered probiotic mixture significantly reduced the logarithmic growth rate of *P. aeruginosa*, decreased inflammatory cytokines, and improved cell viability. This suggests that probiotics can play a meaningful role in moderating the inflammatory response associated with *P. aeruginosa* infections. However, there is limited clinical evidence to definitively confirm that probiotic or postbiotic interventions can improve respiratory health in human populations [57]. Further research is warranted to elucidate the mechanisms by which gut microbiota influence lung diseases and to establish robust clinical applications of these findings.

### 3.6. Lung Cancer

Cancer is a major healthcare challenge. Cancer was the second leading cause of death in the United States in 2023, and lung cancer is the number one cause of cancer death in both men and women [103]. As the gastrointestinal tract and respiratory tract share a common embryonic origin, several similar physiological processes, and structural similarities, the existence of the gut–lung axis has been postulated as an important element in the development of many diseases and potential treatment options [1]. Despite the anatomical remoteness of the lungs and intestines, the organs and their microbiota remain in close contact: indirectly through the lymphatic and circulatory systems and directly through the inhalation of gastro-oesophageal contents and the swallowing of sputum [104]. One way in which the gut microbiome influences oncogenesis in the lungs is that an excess intake of certain substances, e.g., high protein intake, can result in increased protein levels in the colon, where many types of bacteria, including some *Firmicutes* and *Bacteroides* sp., can ferment amino acids to N-nitroso compounds, which induce DNA alkylation and mutations in the host [105]. Through a similar mechanism, metabolites of the gut microbiota, such as reactive oxygen species (ROS), reactive nitrogen species (RNS), and other substances that have proven genotoxic (DNA damaging) effects thus promoting carcinogenesis, appear in the body [106]. Other examples of gut bacterial metabolites are deoxycholic acid and lithocholic acid, which are secondary bile acids produced from bile acids by gut bacteria which cause DNA damage and have been linked to cancer initiation [107]. Disruption of the host microbiome also contributes to the modulation of the host inflammatory response and cell cycle disruption [108]. Indirect evidence for the association of the gut microbiota with lung cancer is also provided by the fact that the use of more antibiotics in a population, which negatively affect the composition of the gut microbiota, correlates positively with the incidence of lung cancer [109]. Cheng et al. showed that commensal bacteria stimulated the Myd88-dependent production of IL-1β and IL-23 from myeloid cells, inducing the proliferation and activation of Vγ6+Vδ1+ γδ T cells, which produced IL-17 and other effector molecules to promote inflammation and tumor cell proliferation [110]. In their work, Zheng et al. even demonstrated a characteristic gut microbial composition leading to a predisposition to lung cancer (richer in *Bacillus* and *Akkermansia muciniphila*) [111]. The same work also identified a microbiota composition that has a ‘protective’ effect (*Bifidobacterium* and *Faecalibacterium*) by, among other things, reducing inflammation induced by TNF-α and LPS [111,112]. TNF-α induces epithelial-to-mesenchymal transition, thereby promoting lung cancer metastasis [113]. In addition, lower loads of *Kluyver*, *Escherichia-Shigella*, *Dialister*, *Faecalibacterium*, and *Enterobacter* have been reported in patients with lung cancer (Table 9) [114]. The results of the above work may thus serve as a potential predictor of early-stage lung cancer. The causal relationship between the gut microbiota and different types of lung cancer has also been the focus of many other studies; for example, using Mendelian randomized genome analysis, Li et al. identified potential causal associations between ten microbial communities and lung cancer, ten with lung adenocarcinoma, nine with lung squamous cell carcinoma, and eleven with small cell lung cancer. After adjustment, *Peptococcaceae* showed a strong causal association with lung adenocarcinoma [115]. Carbohydrates available to the gut microbiota can increase SCFAs which, through T cell receptor signaling, can activate ILC3, producing IL-22, regulatory T cells, and Th2 cells in the lungs, which reduce inflammation and thereby reduce the incidence of lung cancer. The gut microbiota also influences treatment efficacy in lung cancer. For example, oral intake of *Lactobacillus acidophilus* during cisplatin chemotherapy in mouse models of lung cancer has been shown to increase the anti-tumor efficacy of cisplatin, reduce tumor size, and improve survival rates [116]. It has also been shown that an addition of supplementation with the probiotic *Clostridium butyricum* (CBT) to standard ICB (immune checkpoint blockade) therapy before and/or after therapy resulted in significantly a longer progression-free survival in non-small cell lung cancer and a longer overall survival of patients [117]. The gut microbiome has been shown to significantly influence therapy with immune checkpoint inhibitors including those targeting the programmed cell death protein (PD)–1/PD–ligand(L)1 axis. This influence is caused, among other things, by altering the differentiation of regulatory T cells, which further generates changes in immunomodulatory mechanisms. For example, *Akkermansia muciniphila* increased the response to immune checkpoint inhibitor therapy for cancer, while an abnormal composition of the gut microbiota is associated with resistance to the above treatment [118]. In patients with lung cancer, a positive correlation was found between improved response to anti-PD1 therapy and *Akkermansia muciniphila* species abundance [119]. Similarly, *Proteobacteria*, *Firmicutes*, *Bacteroidetes*, and *Actinobacteria* increase the response to anti-PD-1 immunotherapy [120]. Some studies showed that high yogurt intake resulted in a 30% reduction in lung cancer risk, demonstrating that prebiotics and probiotics may have important protective roles in lung carcinogenesis [121]. A single microbial population may not be sufficient to induce or prevent lung cancer, but as a result of a concerted action of the host (such as the immune system), the environment (such as dietary mutagens), or other microorganisms (an amplifying effect), the gut microbiome may exert a carcinogenic effect but also have predictive significance, a protective effect, and enhance or attenuate the therapeutic effect of various forms of therapy [122].

### 3.7. Autoimmune Diseases

Autoimmune diseases, such as rheumatoid arthritis and systemic lupus erythematosus, often involve the gut and lungs together. The gut microbiota can influence systemic autoimmunity, potentially affecting the lungs. Recent studies have suggested that gut dysbiosis may exacerbate autoimmune responses, leading to conditions like interstitial lung disease in patients with systemic autoimmune diseases [17]. The presence of commensal bacteria (such as *Bacteroides fragilis*) in the gut microbiota has been shown to promote a local immune response that prevents autoimmunity in further tissue areas. Rheumatoid arthritis is an example of an autoimmune disease caused by abnormal gut microbiota composition [123] (Table 10). It stimulates the formation of autoreactive T lymphocytes (Th1 and Th12), which move through peripheral immune organs, stimulating B cell differentiation to plasma cells by producing pro-inflammatory cytokines (IL-17, TNF-α, IFN-γ). This leads to the secretion of auto-antibodies, that, together with immune cells, migrate to the synovial tissue, where inflammation is induced through the activation of macrophages, fibroblasts, and osteoclasts, leading to arthritis and psoriasis [123].

## 4. Therapeutic Potential

### 4.1. Probiotics, Prebiotics, and Synbiotics

The demonstrated impact of the gut microbiota on the functioning of the respiratory system and the prevention of the development of many respiratory system diseases has led to increased interest in the use of probiotics (alive pro-health microbial strains), prebiotics (components promoting the growth of probiotics and other healthy microbiota, usually recognized as non-digestible dietary fibers: fructooligosaccharides, galactooligosaccharides, pectins), or synbiotics (combinations of probiotics and prebiotics) against respiratory system disorders and for the modulation of the lung microbiota. The probiotic strains may act directly after aspiration or indirectly through their metabolic products or immune cells activated in the gastrointestinal tract. Probiotic strains exert anti-inflammatory effects through T regulatory cell (Treg) activation, anti-inflammatory cytokine production, stimulation of the Th1 response to allergens, and an enhancement of tolerogenic dendritic cells [52,55,124]. Probiotic strains of the genus *Lactobacillus* and *Bifidobacterium* have generally been shown to reduce lung inflammation However, such a pro-health effect is not always fully confirmed. The controversy includes, for example, the study of patients with cystic fibrosis (CF). Weiss et al., in a prospective pilot study of CF patients (*n* = 10), demonstrated that the number of pulmonary exacerbations was significantly reduced during 6 months of the mixed probiotic (containing *Lactobacillus acidophilus*, *Lactobacillus bulgaricus*, *Bifidobacterium bifidum*, and *Streptococcus thermophiles*) oral intake. Interestingly, there were no changes in pulmonary function tests, sputum bacterial load, neutrophil count, and IL-8 level between treated and control patients [125]. Batoni et al. tested probiotic strains commercially available in Italy (*Lacticaseibacillus rhamnosus*, *L. rhamnosus* ATCC 7469, *L. paracasei*, *Limosilactobacillus fermentum*, *Lactiplantibacillus plantarum*, *Lactobacillus acidophilus*, *L. gasseri)* because of their ability to adhere to the human lung epithelial cell line A549 and inhibit the adhesion of *P. aeruginosa* as one of the major lung pathogens in cystic fibrosis [126]. It was shown that probiotic strains differed in such abilities and *L. acidophilus* displayed the highest adhesion to eukaryotic cells and was the most efficient in preventing the *P. aeruginosa* isolate from adhering to the CF sputum. Moreover, live and UV-killed *L. acidophilus* significantly reduced the amount of pro-inflammatory IL-1β and IL-6 in culture supernatants of peripheral blood mononuclear cells (PBMCs) [126]. On the other hand, Bruzzee et al. did not confirm the pro-health effect of *Lactobacillus rhamnosus* GG (LGG) administration over 12 months on the respiratory and nutritional tracts of children with CF (*n* = 81 including the placebo group). The number of pulmonary exacerbations and hospitalizations was not significantly different between the tested and control (placebo) groups. The authors speculated that earlier interventions, larger doses, or varied strains of probiotics may have an impact [127]. Limited evidence and discrepancies in the efficacy of probiotics, prebiotics, or synbiotics in CF patients prevent the development of specific indications for their use.

### 4.2. Dietary Interventions

Dietary interventions play a pivotal role in modulating the gut microbiome, thereby potentially affecting lung health through the gut–lung axis. One of the most well-established connections between diet, the gut microbiome, and lung health is through the consumption of dietary fiber. Dietary fiber, particularly from fruits, vegetables, whole grains, and legumes, is fermented by gut bacteria to produce short-chain fatty acids (SCFAs), such as acetate, propionate, and butyrate. SCFAs, especially butyrate, can regulate immune responses by promoting the differentiation of Treg cells and suppressing the production of pro-inflammatory cytokines that help reduce inflammation [32]. Studies have shown that higher levels of SCFAs in the gut are associated with better lung function, suggesting that diets rich in fiber could protect against inflammatory lung diseases. Omega-3 fatty acids, primarily found in fatty fish (e.g., salmon, mackerel), flaxseeds, and walnuts, have well-documented anti-inflammatory properties [128]. These fatty acids can reduce the production of inflammatory mediators, such as leukotrienes and prostaglandins, which are implicated in both gut and lung inflammation [128,129]. Dietary intake of omega-3 fatty acids has been associated with a reduced risk of asthma development and severity, likely due to their ability to dampen inflammatory processes within the airways. Omega-3s may also enhance the immune response to respiratory infections by improving macrophage and neutrophil function, thereby preventing excessive inflammation and lung damage [130]. Certain antioxidants, particularly polyphenols, can influence the composition of the gut microbiome [131]. For instance, polyphenols from green tea, grapes, and cocoa have been shown to promote the growth of beneficial bacteria such as *Lactobacillus* and *Bifidobacterium* [132,133]. Vitamin D plays a crucial role in gut and lung health by modulating the immune system [17,28]. This vitamin can enhance gut barrier function, reducing the translocation of harmful bacteria and endotoxins into the bloodstream, which might otherwise trigger systemic and pulmonary inflammation. Adequate vitamin D levels have been associated with a lower risk of respiratory infections, reduced asthma exacerbations, and improved overall lung function [134,135]. Dietary sources of vitamin D include fortified foods, oily fish, and mushrooms, though supplementation is often necessary, especially in regions with limited sunlight.

### 4.3. Fecal Microbiota Transplantation (FMT)

FMT involves transferring fecal material containing a complete, stable fecal microbial community from a healthy donor to a recipient through, e.g., infusion via nasogastric/nasoduodental/nasojejunal tubes, the oral administration of capsules, enemas, or colonoscopies to restore a balanced gut microbiota [136,137,138]. This intervention is used as an experimental treatment for severe, recurrent *Clostridioides difficile* infection (CDI) leading to pseudomembranous colitis, and it has shown promise in patients with inflammatory bowel disease (IBD). FMT significantly lowered the risk of recurrent CDI and caused remission from ulcerative colitis in recipients with IBD by increasing microbial diversity in the intestinal tract and modifying the environment of this microniche (e.g., increased level of SCFAs was noted). Observations of the remission of other diseases (e.g., idiopathic thrombocytopenic purpura, multiple sclerosis) after FMT in patients with ulcerative colitis have led to increased interest in the use of FMT in supporting the treatment of disorders that do not directly involve the digestive system [136,137,139,140,141,142]. FMT may also have potential applications in modulating the gut–lung axis to improve respiratory health. Jang et al. showed that fecal transplants given by oral gavage and high-fiber diets helped to mitigate the lung damage caused in mice exposed to cigarette smoke. FMT attenuated body weight loss and alveolar destruction in emphysema mice. Moreover, IL-6 and IFN-γ levels in bronchoalveolar lavage fluid and serum were lower in both the FMT-treated and high-fiber diet-receiving mice with emphysema compared to non-treated mice indicating the anti-inflammatory effect of FMT and diet [143]. Tang et al. investigated the effect of FMT on pneumonia-derived sepsis caused by *Klebsiella pneumoniae* via the gut–lung axis on the C57BL/6 mice model. FMT restored changes in gut microbes’ diversity induced by pulmonary infection [144]. Both FMT used alone and FMT applied after antibiotic treatment improved animal mortality rates. The antibiotic-treated mice had lower levels of pro-inflammatory cytokines (IL-1β, IL-6, TNF-α) and higher levels of anti-inflammatory IL-10 in comparison to FMT-treated mice, but FMT improved pulmonary local pathological injury more than antibiotics and improved airway epithelial barrier function [144]. In 2023 a multicenter, randomized, double-blind, placebo-controlled study was started to assess the effects of autologous FMT in patients diagnosed with idiopathic pulmonary fibrosis treated with nintedanib [145]. However, judging by the study’s title, the authors expect modification of the negative intestinal effects of the therapy by nintedanib (ameliorate nintedanib-induced diarrhea) rather than changes in the respiratory system.

### 4.4. Pharmaceutical Interventions

Drugs targeting pathways influenced by gut microbiota and their metabolites could be developed to treat respiratory conditions. Pulmonary fibrosis (PF) is a final disorder resulting from inflammatory damage of the alveolar epithelium, the abnormal proliferative transformation of fibroblasts, chemotaxis, and the differentiation of monocytes into monocyte-derived alveolar macrophages, and massive extracellular matrix deposition [30,146]. The high mortality rate, limited treatment effectiveness, dysregulation of immune response, and participation of the lung microbiota and pathogens in the development of PF have become the basis for considering the use of the gut–lung axis in new therapeutic options. Gut microbiota metabolites such as SCFAs and amino acids can regulate immune cell activation, reduce collagen deposition, and inhibit fibroblast differentiation alleviating PF changes. Thus, some pharmaceuticals modulating gut microbiota composition and metabolisms, including natural products such as traditional Chinese medicine (e.g., Qingwen Gupi decoction, Xiao Chai Hu decoction, *Astragalus* polysaccharide), *Amygdalus mongolica* oil, or phycocyanin derived from blue-green algae, are considered as potential new drugs to improve lung conditions in PF [30].

Since SCFAs are the main biologically active metabolites of the gut microbiota, the possibility of using SCFA receptor agonists as potential immunomodulatory drugs in treating gut and lung disorders has also been considered. Theoretically, SCFA receptor agonists might mimic the beneficial effects of SCFAs. However, the results of the studies did not confirm the efficacy of agonists similar to the SCFAs. D’Souza et al. tested the effects of the agonists of GPR43 (G-protein coupled receptor 43), one of the SCFA receptors, within the scope of their potential application in the therapy of IBD [147]. It was found that tested SCFAs (butyrate, propionate, acetate) exhibited a protective effect through the enhancement of intestinal epithelial barrier function and immunoregulatory properties. SCFAs inhibited LPS-induced cytokine production in PBMCs, and human T cell proliferation and cytokine production. Meanwhile, GPR43 agonists failed to tighten the intestinal barrier and exert anti-inflammatory properties, demonstrating much narrower protective functions in IBD than SCFAs [147].

## 5. Conclusions

The gut–lung axis represents a fascinating and complex interplay between two vital systems within the body. Understanding this connection can lead to innovative strategies for preventing and treating respiratory diseases. Future research should focus on unraveling the precise mechanisms and exploring the potential of microbiota-targeted therapies in clinical practice. As research in this field progresses, it holds the potential to revolutionize our approach to health and disease, emphasizing the importance of maintaining a balanced microbiome for both gut and respiratory health.

## Figures and Tables

**Table 1:** Variation in gut microbiota composition in asthma and allergy studies.

Study Population	Microbiota Variation	Key Findings	References
Children (up to 6 years)	Dysbiosis, ↓ *Bacteroides* and ketosphinganine	Asthma development	[67]
Children (3–6 months)	↑ *Firmicutes* and *Clostridia*	Withdrawal of allergies later on	[68]
Children (up to 13 years)	↓ *Bifidobacterium*	Allergic rhinitis	[70]
Mouse model	Administration of probiotics containing *Lachnospira*, *Veillonella*, *Faecalibacterium* and *Rothia*	Mitigation of airway inflammation	[69]

**Table 2:** Variation in gut microbiota composition in COPD studies.

Study Population	Microbiota Variation	Key Findings	References
COPD patients	↑ *Faecalicatena*, *Oscillibacter*, *Lawsonibacter*, *Flavonifractor*, and *Streptomyces*, ↓ *Lachnospira*, *Eubacterium*, and *Coprococcus*	COPD severity	[78]
Mouse model	↓ *Erysipelotrichaceae*, *Bacteroidales*, and *Ruminococcaceae*, ↑ *Lachnospiriaceae*	Accompanying cigarette smoking-induced COPD (CS-COPD)	[77]

**Table 3:** Variation in gut microbiota composition in TB studies.

Study Population	Microbiota Variation	Key Findings	References
TB patients	↓ *Firmicutes*, *Proteobacteria* and *Verrucomicrobia*, ↑ *Actinobacteria*, *Bacteroidetes*, and *Fusobacteria*	*Mtb* infection dysregulates immune system and alters gut microbiome	[83]
TB patients	↓ *Bacteroides*, *Prevotella* and *Lachnospira*, ↑ *Actinobacteria* and *Proteobacteria*	Accompanying new and recurrent TB	[84]
Children with TB	↓ *Actinobacteria*, *Bifidobacterium*, *Dorea*, *Faecalibacterium*, and *Ruminococcus*, ↑ *Bacteroides*, *Proteobacteria*, *Enterococcus* and *Prevotella*	Correlation with TB progression	[85]

**Table 4:** Variation in gut microbiota composition in COVID-19 studies.

Study Population	Microbiota Variation	Key Findings	References
COVID-19 patients	↓ *Bifidobacterium*, *Faecalibacterium*, and other butyrate producers, ↑ *Streptococcus*, *Actinomyces*, and *Rothia*	COVID-19 is accompanied by gut dysbiosis; gut microbiota regulates ACE2 receptor expression in colon	[82,87,88]
ICU patients with SARS-CoV-2 pneumonia	Administration of probiotics containing *Lactobacillus*, *Bifidobacterium* and *Streptococcus*	Reduced mortality	[82,93,94,95]
Critically ill patients with SARS-CoV-2 pneumonia	Oral administration of *Lactobacillus helveticus*	Modulation of the immune system including an increased number of IgA-secreting cells in the gut and broncho-associated lymphoid tissue	[89]
COVID-19 patients	Administration of synbiotics consisting of *Bifidobacterium* strains and prebiotics (SIM01)	Resolution of COVID-19 clinical symptoms, ↑ IgG to SARS-CoV-2, ↓ blood pro-inflammatory markers (IL-6, CCL2, M-CSF, TNF, IL-1RA)	[87]

**Table 5:** Modification of gut microbiota composition in influenza studies.

Study Population	Microbiota Variation	Key Findings	References
Mouse model	Oral administration of 10 lactic acid bacteria (LAB) strains	Mitigation of influenza symptoms through immunomodulatory effects	[96]
Mouse model	Oral administration *of Bifidobacterium longum* MM-2 isolate to influenza-infected mice	↓ Inflammatory responses in the lower respiratory tract and animal mortality by immunomodulatory effects	[91]
Mouse model	Intranasal or oral administration of *L. plantarum DK119*	Protects against a lethal dose of influenza A virus by immunomodulatory effects	[91]
Mouse model	Oral administration of *L. rhamnosus* M21	↓ Inflammatory damage in the lungs and ↑ IFN-γ and IL-2 levels in lung lysates	[97]
Mouse model	Oral administration of probiotic containing *L. mucosae* 1025 and *B. breve* CCFM1026	↓ Stool butyrate level and attenuates inflammation in lungs	[97]
Children (2–6 years)	Daily consumption of a milk drink containing *L. rhamnosus* GG	No effect on the incidence of various respiratory viral infections, including influenza virus, and respiratory symptoms	[89]

**Table 6:** Modification of gut microbiota composition in RSV studies.

Study Population	Microbiota Variation	Key Findings	References
Mouse model	Oral administration of probiotics containing *Lactobacillus*, *Bifidobacterium*, *Enterococcus* or *Lactococcus*	↓ Symptoms of later RSV infection and improved survival	[82]

**Table 7:** Variation in gut microbiota composition in *S. pneumoniae* lung infections.

Study Population	Microbiota Variation	Key Findings	References
Mouse model	Post-antibiotic dysbiosis	↓ Mortality because of subsequent *S. pneumoniae* infection, ↓ levels of pro-inflammatory cytokines, impairment of alveolar macrophage functions	[100]
Mouse model	Intranasal administration of probiotocs	↑ Local production of TNF-α and IFN-γ, ↓ tissue damage	[101]

**Table 8:** Modification of gut microbiota composition in *P. aeruginosa* lung infections.

Study Population	Microbiota Variation	Key Findings	References
Mouse model	Endotracheal instillation of probiotic containing *Lactobacillus fermentum*, *L. paraesei*, and *L. zeae*	↓ Logarithmic growth rate of *P. aeruginosa* and levels of inflammatory cytokines, ↑ cell viability	[57]

**Table 9:** Variation and modification in gut microbiota composition in lung cancer studies.

Study Population	Microbiota Variation	Key Findings	References
Lung cancer patients	↑ *Bacillus* and *Akkermansia muciniphila*	Predisposition to lung cancer development	[111]
Lung cancer patients	↑ *Bifidobacterium* and *Faecalibacterium*	Protection against lung cancer by reducing inflammation	[111,112]
Mouse model	Oral intake of *Lactobacillus acidophilus*	↑ Anti-tumor efficacy of cisplatin, ↓ tumor size, improved survival rates	[116]
Lung cancer patients	Supplementation of probiotic *Clostridium butyricum* strain before and/or after standard ICB therapy	Longer progression-free survival of non-small cell lung cancer and overall survival of patients	[117]
Lung cancer patients	High yogurt intake	30% reduction in lung cancer risk	[121]

**Table 10:** Variation in microbiota composition in autoimmune diseases studies.

Study Population	Microbiota Variation	Key Findings	Reference
Rheumatoid arthritis patients	Segmented filamentous bacteria induce activation of Th17 cells	Dysbiosis stimulates pro-inflammatory response	[123]

## Data Availability

The data presented in this study are openly available under reference numbers.

## References

[1] Enaud R., Prevel R., Ciarlo E., Beaufils F., Wieërs G., Guery B., Delhaes L. (2020). The Gut-Lung Axis in Health and Respiratory Diseases: A Place for Inter-Organ and Inter-Kingdom Crosstalks. Front. Cell. Infect. Microbiol..

[2] Krajmalnik-Brown R., Ilhan Z.E., Kang D.W., DiBaise J.K. (2012). Effects of gut microbes on nutrient absorption and energy regulation. Nutr. Clin. Pract..

[3] Rowland I., Gibson G., Heinken A., Scott K., Swann J., Thiele I., Tuohy K. (2018). Gut microbiota functions: Metabolism of nutrients and other food components. Eur. J. Nutr..

[4] den Besten G., van Eunen K., Groen A.K., Venema K., Reijngoud D.J., Bakker B.M. (2013). The role of short-chain fatty acids in the interplay between diet, gut microbiota, and host energy metabolism. J. Lipid Res..

[5] Budden K.F., Gellatly S.L., Wood D.L., Cooper M.A., Morrison M., Hugenholtz P., Hansbro P.M. (2017). Emerging pathogenic links between microbiota and the gut-lung axis. Nat. Rev. Microbiol..

[6] Cheng H., Ning M., Chen D., Ma W. (2019). Interactions Between the Gut Microbiota and the Host Innate Immune Response Against Pathogens. Front. Immunol..

[7] Belkaid Y., Harrison O.J. (2017). Homeostatic Immunity and the Microbiota. Immunity.

[8] Haldar S., Jadhav S.R., Gulati V., Beale D.J., Balkrishna A., Varshney A., Palombo E.A., Karpe A.V., Shah R.M. (2023). Unravelling the gut-lung axis: Insights into microbiome interactions and Traditional Indian Medicine’s perspective on optimal health. FEMS Microbiol. Ecol..

[9] Belkaid Y., Hand T. (2014). Role of the microbiota in immunity and inflammation. Cell.

[10] Li R., Li J., Zhou X. (2024). Lung microbiome: New insights into the pathogenesis of respiratory diseases. Signal Transduct. Target. Ther..

[11] Cho I., Blaser M.J. (2012). The human microbiome: At the interface of health and disease. Nat. Rev. Genet..

[12] Ley R.E., Turnbaugh P.J., Klein S., Gordon J.I. (2006). Microbial ecology: Human gut microbes associated with obesity. Nature.

[13] Sze M.A., Dimitriu P.A., Hayashi S., Elliott W.M., McDonough J.E., Gosselink J.V., Cooper J., Sin D.D., Mohn W.W., Hogg J.C. (2012). The lung tissue microbiome in chronic obstructive pulmonary disease. Am. J. Respir. Crit. Care Med..

[14] Rinninella E., Raoul P., Cintoni M., Franceschi F., Miggiano G.A.D., Gasbarrini A., Mele M.C. (2019). What is the healthy gut microbiota composition? A changing ecosystem across age, environment, diet, and diseases. Microorganisms.

[15] Manor O., Levy R., Pope C.E., Hayden H.S., Brittnacher M.J., Carr R., Radey M.C., Hager K.R., Heltshe S.L., Ramsey B.W. (2016). Metagenomic evidence for taxonomic dysbiosis and functional imbalance in the gastrointestinal tracts of children with cystic fibrosis. Sci. Rep..

[16] Al Bander Z., Nitert M.D., Mousa A., Naderpoor N. (2020). The Gut Microbiota and Inflammation: An Overview. Int. J. Environ. Res. Public Health.

[17] Eladham M.W., Selvakumar B., Saheb Sharif-Askari N., Saheb Sharif-Askari F., Ibrahim S.M., Halwani R. (2024). Unraveling the gut-Lung axis: Exploring complex mechanisms in disease interplay. Heliyon.

[18] Tang J., Xu L., Zeng Y., Gong F. (2021). Effect of gut microbiota on LPS-induced acute lung injury by regulating the TLR4/NF-kB signaling pathway. Int. Immunopharmacol..

[19] Yip W., Hughes M.R., Li Y., Cait A., Hirst M., Mohn W.W., McNagny K.M. (2021). Butyrate shapes immune cell fate and function in allergic asthma. Front. Immunol..

[20] Bradley C.P., Teng F., Felix K.M., Sano T., Naskar D., Block K.E., Huang H., Knox K.S., Littman D.R., Wu H.J. (2017). Segmented Filamentous Bacteria provoke lung autoimmunity by inducing gut-lung axis Th17 cells expressing dual TCRs. Cell Host Microbe.

[21] Jonsson H., Hugerth L.W., Sundh J., Lundin E., Andersson A.F. (2020). Genome sequence of segmented filamentous bacteria present in the human intestine. Commun. Biol..

[22] McQuade R.M., Bandara M., Diwakarla S., Sahakian L., Han M.N., Al Thaalibi M., Di Natale M.R., Tan M., Harwood K.H., Schneider-Futschik E.K. (2023). Gastrointestinal consequences of lipopolysaccharide-induced lung inflammation. Inflamm. Res..

[23] Goossens E., Li J., Callens C., Van Rysselberghe N., Kettunen H., Vuorenmaa J., Garcia Gonzalez N., Libert C., Ducatelle R., Van Immerseel F. (2022). Acute endotoxemia-induced respiratory and intestinal dysbiosis. Int. J. Mol. Sci..

[24] Lane S., Hilliam Y., Bomberger J.M. (2023). Microbial and immune regulation of the gut-lung axis during viral-bacterial coinfection. J. Bacteriol..

[25] Campbell C., Kandalgaonkar M.R., Golonka R.M., Yeoh B.S., Vijay-Kumar M., Saha P. (2023). Crosstalk between Gut Microbiota and Host Immunity: Impact on Inflammation and Immunotherapy. Biomedicines.

[26] Espírito Santo C., Caseiro C., Martins M.J., Monteiro R., Brandão I. (2021). Gut Microbiota, in the Halfway between Nutrition and Lung Function. Nutrients.

[27] Guo J., Wang L., Han N., Yuan C., Yin Y., Wang T., Sun J., Jin P., Liu Y., Jia Z. (2024). People are an organic unity: Gut-lung axis and pneumonia. Heliyon.

[28] Anand S., Mande S.S. (2018). Diet, Microbiota and Gut-Lung Connection. Front. Microbiol..

[29] Dang A.T., Marsland B.J. (2019). Microbes, metabolites, and the gut–lung axis. Mucosal Immunol..

[30] Dong Y., He L., Zhu Z., Yang F., Ma Q., Zhang Y., Zhang X., Liu X. (2024). The mechanism of gut-lung axis in pulmonary fibrosis. Front. Cell. Infect. Microbiol..

[31] Li Z., Li Y., Sun Q., Wei J., Li B., Qiu Y., Liu K., Shao D., Ma Z. (2022). Targeting the Pulmonary Microbiota to Fight against Respiratory Diseases. Cells.

[32] Verma A., Bhagchandani T., Rai A., Nikita Sardarni U.K., Bhavesh N.S., Gulati S., Malik R., Tandon R. (2024). Short-Chain Fatty Acid (SCFA) as a Connecting Link between Microbiota and Gut-Lung Axis-A Potential Therapeutic Intervention to Improve Lung Health. ACS Omega.

[33] Blaak E.E., Canfora E.E., Theis S., Frost G., Groen A.K., Mithieux G., Nauta A., Scott K., Stahl B., van Harsselaar J. (2020). Short chain fatty acids in human gut and metabolic health. Benef. Microbes.

[34] Han X., Ma Y., Ding S., Fang J., Liu G. (2023). Regulation of dietary fiber on intestinal microorganisms and its effects on animal health. Anim. Nutr..

[35] Trompette A., Gollwitzer E.S., Yadava K., Sichelstiel A.K., Sprenger N., Ngom-Bru C., Blanchard C., Junt T., Nicod L.P., Harris N.L. (2014). Gut microbiota metabolism of dietary fiber influences allergic airway disease and hematopoiesis. Nat. Med..

[36] Gauguet S., D’Ortona S., Ahnger-Pier K., Duan B., Surana N.K., Lu R., Cywes-Bentley C., Gadjeva M., Shan Q., Priebe G.P. (2015). Intestinal Microbiota of Mice Influences Resistance to *Staphylococcus aureus* Pneumonia. Infect. Immun..

[37] McAleer J.P., Nguyen N.L., Chen K., Kumar P., Ricks D.M., Binnie M., Armentrout R.A., Pociask D.A., Hein A., Yu A. (2016). Pulmonary Th17 Antifungal Immunity Is Regulated by the Gut Microbiome. J. Immunol..

[38] Negi S., Pahari S., Bashir H., Agrewala J.N. (2019). Gut Microbiota Regulates Mincle Mediated Activation of Lung Dendritic Cells to Protect Against *Mycobacterium tuberculosis*. Front. Immunol..

[39] Ancona G., Alagna L., Alteri C., Palomba E., Tonizzo A., Pastena A., Muscatello A., Gori A., Bandera A. (2023). Gut and airway microbiota dysbiosis and their role in COVID-19 and long-COVID. Front. Immunol..

[40] Chakraborty C., Sharma A.R., Bhattacharya M., Dhama K., Lee S.S. (2022). Altered gut microbiota patterns in COVID-19: Markers for inflammation and disease severity. World J. Gastroenterol..

[41] Tieu V., Tibi S., Ling J. (2023). Regulation of SARS-CoV-2 infection by diet-modulated gut microbiota. Front. Cell. Infect. Microbiol..

[42] Zhang F., Lau R.I., Liu Q., Su Q., Chan F.K.L., Ng S.C. (2023). Gut microbiota in COVID-19: Key microbial changes, potential mechanisms and clinical applications. Nat. Rev. Gastroenterol. Hepatol..

[43] Luqman A., Hassan A., Ullah M., Naseem S., Ullah M., Zhang L., Din A.U., Ullah K., Ahmad W., Wang G. (2024). Role of the intestinal microbiome and its therapeutic intervention in cardiovascular disorder. Front. Immunol..

[44] Pedroza Matute S., Iyavoo S. (2023). Exploring the gut microbiota: Lifestyle choices, disease associations, and personal genomics. Front. Nutr..

[45] Hu J., Nomura Y., Bashir A., Fernandez-Hernandez H., Itzkowitz S., Pei Z., Stone J., Loudon H., Peter I. (2013). Diversified microbiota of meconium is affected by maternal diabetes status. PLoS ONE.

[46] He Q., Kwok L.Y., Xi X., Zhong Z., Ma T., Xu H., Meng H., Zhao F., Zhang H. (2020). The meconium microbiota shares more features with the amniotic fluid microbiota than the maternal fecal and vaginal microbiota. Gut Microbes.

[47] Rodríguez J.M., Murphy K., Stanton C., Ross R.P., Kober O.I., Juge N., Avershina E., Rudi K., Narbad A., Jenmalm M.C. (2015). The composition of the gut microbiota throughout life, with an emphasis on early life. Microb. Ecol. Health Dis..

[48] Turunen J., Tejesvi M.V., Paalanne N., Hekkala J., Lindgren O., Kaakinen M., Pokka T., Kaisanlahti A., Reunanen J., Tapiainen T. (2021). Presence of distinctive microbiome in the first-pass meconium of newborn infants. Sci. Rep..

[49] Roager H.M., Stanton C., Hall L.J. (2023). Microbial metabolites as modulators of the infant gut microbiome and host-microbial interactions in early life. Gut Microbes.

[50] Olvera-Rosales L.B., Cruz-Guerrero A.E., Ramírez-Moreno E., Quintero-Lira A., Contreras-López E., Jaimez-Ordaz J., Castañeda-Ovando A., Añorve-Morga J., Calderón-Ramos Z.G., Arias-Rico J. (2021). Impact of the Gut Microbiota Balance on the Health–Disease Relationship: The Importance of Consuming Probiotics and Prebiotics. Foods.

[51] Yang Q., Liang Q., Balakrishnan B., Belobrajdic D.P., Feng Q.J., Zhang W. (2020). Role of Dietary Nutrients in the Modulation of Gut Microbiota: A Narrative Review. Nutrients.

[52] Saeed N.K., Al-Beltagi M., Bediwy A.S., El-Sawaf Y., Toema O. (2022). Gut microbiota in various childhood disorders: Implication and indications. World J. Gastroenterol..

[53] Yang D., Xing Y., Song X., Qian Y. (2020). The impact of lung microbiota dysbiosis on inflammation. Immunology.

[54] Leitao Filho F.S., Monica Peters C., Sheel A.W., Yang J., Nislow C., Lam S., Leung J.M., Sin D.D. (2023). Characterization of the Lower Airways and Oral Microbiota in Healthy Young Persons in the Community. Biomedicines.

[55] Mazziotta C., Tognon M., Martini F., Torreggiani E., Rotondo J.C. (2023). Probiotics Mechanism of Action on Immune Cells and Beneficial Effects on Human Health. Cells.

[56] Cauwenberghs E., De Boeck I., Spacova I., Van Tente I., Bastiaenssen J., Lammertyn E., Verhulst S., Van Hoorenbeeck K., Lebeer S. (2024). Positioning the preventive potential of microbiome treatments for cystic fibrosis in the context of current therapies. Cell Rep. Med..

[57] Mindt B.C., DiGiandomenico A. (2022). Microbiome Modulation as a Novel Strategy to Treat and Prevent Respiratory Infections. Antibiotics.

[58] Lu Y., Zhang Y., Zhao X., Shang C., Xiang M., Li L., Cui X. (2022). Microbiota-derived short-chain fatty acids: Implications for cardiovascular and metabolic disease. Front. Cardiovasc. Med..

[59] Cait A., Hughes M.R., Antignano F., Cait J., Dimitriu P.A., Maas K.R., Reynolds L.A., Hacker L., Mohr J., Finlay B.B. (2018). Microbiome-driven allergic lung inflammation is ameliorated by short-chain fatty acids. Mucosal Immunol..

[60] Thorburn A.N., McKenzie C.I., Shen S., Stanley D., Macia L., Mason L.J., Roberts L.K., Wong C.H., Shim R., Robert R. (2015). Evidence that asthma is a developmental origin disease influenced by maternal diet and bacterial metabolites. Nat. Commun..

[61] Trompette A., Gollwitzer E.S., Pattaroni C., Lopez-Mejia I.C., Riva E., Pernot J., Ubags N., Fajas L., Nicod L.P., Marsland B.J. (2018). Dietary Fiber Confers Protection against Flu by Shaping Ly6c- Patrolling Monocyte Hematopoiesis and CD8+ T Cell Metabolism. Immunity.

[62] Han Y., Wang B., Gao H., He C., Hua R., Liang C., Zhang S., Wang Y., Xin S., Xu J. (2022). Vagus Nerve and Underlying Impact on the Gut Microbiota-Brain Axis in Behavior and Neurodegenerative Diseases. J. Inflamm. Res..

[63] Godlewska U., Bulanda E., Wypych T.P. (2022). Bile acids in immunity: Bidirectional mediators between the host and the microbiota. Front. Immunol..

[64] Kumar H., Kawai T., Akira S. (2011). Pathogen recognition by the innate immune system. Int. Rev. Immunol..

[65] Jeong E., Lee J.Y. (2011). Intrinsic and extrinsic regulation of innate immune receptors. Yonsei Med. J..

[66] Zhou A., Lei Y., Tang L., Hu S., Yang M., Wu L., Yang S., Tang B. (2021). Gut microbiota: The emerging link to lung homeostasis and disease. J. Bacteriol..

[67] Lee-Sarwar K.A., Chen Y.C., Chen Y.Y., Kozyrskyj A.L., Mandhane P.J., Turvey S.E., Subbarao P., Bisgaard H., Stokholm J., Chawes B. (2023). The maternal prenatal and offspring early-life gut microbiome of childhood asthma phenotypes. Allergy.

[68] Arrieta M.C., Stiemsma L.T., Dimitriu P.A., Thorson L., Russell S., Yurist-Doutsch S., Kuzeljevic B., Gold M.J., Britton H.M., Lefebvre D.L. (2015). Early infancy microbial and metabolic alterations affect risk of childhood asthma. Sci. Transl. Med..

[69] Bunyavanich S., Shen N., Grishin A., Wood R., Burks W., Dawson P., Jones S.M., Leung D.Y., Sampson H., Sicherer S. (2016). Early-life gut microbiome composition and milk allergy resolution. J. Allergy Clin. Immunol..

[70] Kallio S., Jian C., Korpela K., Kukkonen A.K., Salonen A., Savilahti E., Kuitunen M., de Vos W.M. (2024). Early-life gut microbiota associates with allergic rhinitis during 13-year follow-up in a Finnish probiotic intervention cohort. Microbiol. Spectr..

[71] Barnes P.J. (2015). Therapeutic approaches to asthma-chronic obstructive pulmonary disease overlap syndromes. J. Allergy Clin. Immunol..

[72] Mirza S., Benzo R. (2017). Chronic obstructive pulmonary disease phenotypes: Implications for care. Mayo Clin. Proc..

[73] Rodrigues S.O., Cunha C.M.C.D., Soares G.M.V., Silva P.L., Silva A.R., Gonçalves-de-Albuquerque C.F. (2021). Mechanisms, pathophysiology and currently proposed treatments of Chronic Obstructive Pulmonary Disease. Pharmaceuticals.

[74] Lange P., Ahmed E., Lahmar Z.M., Martinez F.J., Bourdin A. (2021). Natural history and mechanisms of COPD. Respirology.

[75] Wang Z., Bafadhel M., Haldar K., Spivak A., Mayhew D., Miller B.E., Tal-Singer R., Johnston S.L., Ramsheh M.Y., Barer M.R. (2016). Lung microbiome dynamics in COPD exacerbations. Eur. Respir. J..

[76] GOLD (Global Initiative for Chronic Obstructive Lung Disease) Report. https://goldcopd.org/2024-gold-report/.

[77] Lai H., Lin T., Chen T., Kuo Y.L., Chang C.J., Wu T.R., Shu C.C., Tsai Y.H., Swift S., Lu C.C. (2022). Gut microbiota modulates COPD pathogenesis: Role of anti-inflammatory *Parabacteroides goldsteinii* lipopolysaccharide. Gut.

[78] Liu Y., Teo S.M., Méric G., Tang H.H.F., Zhu Q., Sanders J.G., Vázquez-Baeza Y., Verspoor K., Vartiainen V.A., Jousilahti P. (2023). The gut microbiome is a significant risk factor for future chronic lung disease. J. Allergy Clin. Immunol..

[79] Yu Z., Shen X., Wang A., Hu C., Chen J. (2023). The gut microbiome: A line of defense against tuberculosis development. Front. Cell. Infect. Microbiol..

[80] Comberiati P., Di Cicco M., Paravati F., Pelosi U., Di Gangi A., Arasi S., Barni S., Caimmi D., Mastrorilli C., Licari A. (2021). The Role of Gut and Lung Microbiota in Susceptibility to Tuberculosis. Int. J. Environ. Res. Public Health.

[81] Yuan Z., Kang Y., Mo C., Huang S., Qin F., Zhang J., Wang F., Jiang J., Yang X., Liang H. (2024). Causal relationship between gut microbiota and tuberculosis: A bidirectional two-sample Mendelian randomization analysis. Respir. Res..

[82] Marrella V., Nicchiotti F., Cassani B. (2024). Microbiota and Immunity during Respiratory Infections: Lung and Gut Affair. Int. J. Mol. Sci..

[83] Nguyen M., Ahn P., Dawi J., Gargaloyan A., Kiriaki A., Shou T., Wu K., Yazdan K., Venketaraman V. (2024). The Interplay between *Mycobacterium tuberculosis* and Human Microbiome. Clin. Pract..

[84] Luo M., Liu Y., Wu P., Luo D.X., Sun Q., Zheng H., Hu R., Pandol S.J., Li Q.F., Han Y.P. (2017). Alternation of Gut Micro-biota in Patients with Pulmonary Tuberculosis. Front. Physiol..

[85] Chen L., Zhang G., Li G., Wang W., Ge Z., Yang Y., He X., Liu Z., Zhang Z., Mai Q. (2022). Ifnar gene variants influence gut microbial production of palmitoleic acid and host immune responses to tuberculosis. Nat. Metab..

[86] Dumas A., Bernard L., Poquet Y., Lugo-Villarino G., Neyrolles O. (2018). The role of the lung microbiota and the gut-lung axis in respiratory infectious diseases. Cell. Microbiol..

[87] Zuo T., Zhang F., Lui G.C.Y., Yeoh Y.K., Li A.Y.L., Zhan H., Wan Y., Chung A.C.K., Cheung C.P., Chen N. (2020). Alterations in Gut Microbiota of Patients with COVID-19 during Time of Hospitalization. Gastroenterology.

[88] Aishwarya S., Gunasekaran K., Anita Margret A. (2022). Intermodulation of gut-lung axis microbiome and the implications of biotics to combat COVID-19. J. Biomol. Struct. Dyn..

[89] Shahbazi R., Yasavoli-Sharahi H., Alsadi N., Ismail N., Matar C. (2020). Probiotics in Treatment of Viral Respiratory Infections and Neuroinflammatory Disorders. Molecules.

[90] Effenberger M., Grabherr F., Mayr L., Schwaerzler J., Nairz M., Seifert M., Hilbe R., Seiwald S., Scholl-Buergi S., Fritsche G. (2020). Faecal calprotectin indicates intestinal inflammation in COVID-19. Gut.

[91] Ahmadi Badi S., Tarashi S., Fateh A., Rohani P., Masotti A., Siadat S.D. (2021). From the Role of Microbiota in Gut-Lung Axis to SARS-CoV-2 Pathogenesis. Mediat. Inflamm..

[92] Yuksel N., Gelmez B., Yildiz-Pekoz A. (2023). Lung Microbiota: Its Relationship to Respiratory System Diseases and Approaches for Lung-Targeted Probiotic Bacteria Delivery. Mol. Pharm..

[93] Ceccarelli G., Marazzato M., Celani L., Lombardi F., Piccirilli A., Mancone M., Trinchieri V., Pugliese F., Mastroianni C.M., d’Ettorre G. (2021). Oxygen Sparing Effect of Bacteriotherapy in COVID-19. Nutrients.

[94] Shi Y., Yamazaki T., Okubo Y., Uehara Y., Sugane K., Agematsu K. (2005). Regulation of aged humoral immune defense against pneumococcal bacteria by IgM memory B cell. J. Immunol..

[95] Waki N., Matsumoto M., Fukui Y., Suganuma H. (2014). Effects of probiotic Lactobacillus brevis KB290 on incidence of influenza infection among schoolchildren: An open-label pilot study. Lett. Appl. Microbiol..

[96] Takeda S., Takeshita M., Kikuchi Y., Dashnyam B., Kawahara S., Yoshida H., Watanabe W., Muguruma M., Kurokawa M. (2011). Efficacy of oral administration of heat-killed probiotics from Mongolian dairy products against influenza infection in mice: Alleviation of influenza infection by its immunomodulatory activity through intestinal immunity. Int. Immunopharmacol..

[97] Ou G., Xu H., Wu J., Wang S., Chen Y., Deng L., Chen X. (2023). The gut-lung axis in influenza A: The role of gut microbiota in immune balance. Front. Immunol..

[98] Rastogi S., Mohanty S., Sharma S., Tripathi P. (2022). Possible role of gut microbes and host’s immune response in gut-lung homeostasis. Front. Immunol..

[99] Sencio V., Machado M.G., Trottein F. (2021). The lung-gut axis during viral respiratory infections: The impact of gut dysbiosis on secondary disease outcomes. Mucosal Immunol..

[100] Schuijt T.J., Lankelma J.M., Scicluna B.P., de Sousa e Melo F., Roelofs J.J., de Boer J.D., Hoogendijk A.J., de Beer R., de Vos A., Belzer C. (2016). The gut microbiota plays a protective role in the host defence against pneumococcal pneumonia. Gut.

[101] Hori T., Kiyoshima J., Shida K., Yasui H. (2001). Effect of intranasal administration of *Lactobacillus casei* Shirota on influenza virus infection of upper respiratory tract in mice. Clin. Diagn. Lab. Immunol..

[102] Alexandre Y., Le Berre R., Barbier G., Le Blay G. (2014). Screening of *Lactobacillus* spp. for the prevention of Pseudomonas aeruginosa pulmonary infections. BMC Microbiol..

[103] Siegel R.L., Miller K.D., Wagle N.S., Jemal A. (2023). Cancer Statistics, 2023. CA Cancer J. Clin..

[104] Renz H., Brandtzaeg P., Hornef M. (2012). The Impact of Perinatal Immune Development on Mucosal Homeostasis and Chronic Inflammation. Nat. Rev. Immunol..

[105] Gill C.I.R., Rowland I.R. (2002). Diet and Cancer: Assessing the Risk. Br. J. Nutr..

[106] Devkota S., Wang Y., Musch M.W., Leone V., Fehlner-Peach H., Nadimpalli A., Antonopoulos D.A., Jabri B., Chang E.B. (2012). Dietary-Fat-Induced Taurocholic Acid Promotes Pathobiont Expansion and Colitis in Il10−/− Mice. Nature.

[107] Louis P., Hold G.L., Flint H.J. (2014). The Gut Microbiota, Bacterial Metabolites and Colorectal Cancer. Nat. Rev. Microbiol..

[108] Mao Q., Jiang F., Yin R., Wang J., Xia W., Dong G., Ma W., Yang Y., Xu L., Hu J. (2018). Interplay between the Lung Microbiome and Lung Cancer. Cancer Lett..

[109] Boursi B., Mamtani R., Haynes K., Yang Y.-X. (2015). Recurrent Antibiotic Exposure May Promote Cancer Formation—Another Step in Understanding the Role of the Human Microbiota?. Eur. J. Cancer.

[110] Jin C., Lagoudas G.K., Zhao C., Bullman S., Bhutkar A., Hu B., Ameh S., Sandel D., Liang X.S., Mazzilli S. (2019). Commensal Microbiota Promote Lung Cancer Development via Γδ T Cells. Cell.

[111] Zheng Y., Fang Z., Xue Y., Zhang J., Zhu J., Gao R., Yao S., Ye Y., Wang S., Lin C. (2020). Specific Gut Microbiome Signature Predicts the Early-Stage Lung Cancer. Gut Microbes.

[112] Boesten R., Schuren F., Willemsen L., Vriesema A., Knol J., De Vos W. (2011). *Bifidobacterium Breve*–HT-29 Cell Line Interaction: Modulation of TNF-α Induced Gene Expression. Benef. Microbes.

[113] Shang G.-S., Liu L., Qin Y.-W. (2017). IL-6 and TNF-α Promote Metastasis of Lung Cancer by Inducing Epithelial-Mesenchymal Transition. Oncol. Lett..

[114] Zhang W.-Q., Zhao S.-K., Luo J.-W., Dong X.-P., Hao Y.-T., Li H., Shan L., Zhou Y., Shi H.-B., Zhang Z.-Y. (2018). Alterations of Fecal Bacterial Communities in Patients with Lung Cancer. Am. J. Transl. Res..

[115] Li Y., Wang K., Zhang Y., Yang J., Wu Y., Zhao M. (2023). Revealing a Causal Relationship between Gut Microbiota and Lung Cancer: A Mendelian Randomization Study. Front. Cell. Infect. Microbiol..

[116] Gui Q.-F., Lu H.-F., Zhang C.-X., Xu Z.-R., Yang Y.-H. (2015). Well-Balanced Commensal Microbiota Contributes to Anti-Cancer Response in a Lung Cancer Mouse Model. Gen. Mol. Res..

[117] Tomita Y., Ikeda T., Sakata S., Saruwatari K., Sato R., Iyama S., Jodai T., Akaike K., Ishizuka S., Saeki S. (2020). Association of Probiotic Clostridium Butyricum Therapy with Survival and Response to Immune Checkpoint Blockade in Patients with Lung Cancer. Cancer Immunol. Res..

[118] Routy B., Le Chatelier E., Derosa L., Duong C.P.M., Alou M.T., Daillère R., Fluckiger A., Messaoudene M., Rauber C., Roberti M.P. (2018). Gut Microbiome Influences Efficacy of PD-1–Based Immunotherapy against Epithelial Tumors. Science.

[119] Zhao Y., Liu Y., Li S., Peng Z., Liu X., Chen J., Zheng X. (2021). Role of Lung and Gut Microbiota on Lung Cancer Pathogenesis. J. Cancer Res. Clin. Oncol..

[120] Song P., Yang D., Wang H., Cui X., Si X., Zhang X., Zhang L. (2020). Relationship between Intestinal Flora Structure and Metabolite Analysis and Immunotherapy Efficacy in Chinese NSCLC Patients. Thorac. Cancer.

[121] Yang J.J., Yu D., Xiang Y.-B., Blot W., White E., Robien K., Sinha R., Park Y., Takata Y., Lazovich D. (2020). Association of Dietary Fiber and Yogurt Consumption with Lung Cancer Risk. JAMA Oncol..

[122] Teng J., Zhao Y., Jiang Y., Wang Q., Zhang Y. (2020). Correlation between Gut Microbiota and Lung Cancer. Zhongguo Fei Ai Za Zhi.

[123] Scher J.U., Abramson S.B. (2011). The microbiome and rheumatoid arthritis. Nat. Rev. Rheumatol..

[124] Sestito S., D’Auria E., Baldassarre M.E., Salvatore S., Tallarico V., Stefanelli E., Tarsitano F., Concolino D., Pensabene L. (2020). The Role of Prebiotics and Probiotics in Prevention of Allergic Diseases in Infants. Front. Pediatr..

[125] Weiss B., Bujanover Y., Yahav Y., Vilozni D., Fireman E., Efrati O. (2010). Probiotic supplementation affects pulmonary exacerbations in patients with cystic fibrosis: A pilot study. Pediatr. Pulmonol..

[126] Batoni G., Kaya E., Catelli E., Quinti S., Botti M., De Carli A., Bianchi M., Maisetta G., Esin S. (2023). Lactobacillus Probiotic Strains Differ in Their Ability to Adhere to Human Lung Epithelial Cells and to Prevent Adhesion of Clinical Isolates of Pseudomonas aeruginosa from Cystic Fibrosis Lung. Microorganisms.

[127] Bruzzese E., Raia V., Spagnuolo M.I., Volpicelli M., De Marco G., Maiuri L., Guarino A. (2007). Effect of Lactobacillus GG supplementation on pulmonary exacerbations in patients with cystic fibrosis: A pilot study. Clin. Nutr..

[128] Costantini L., Molinari R., Farinon B., Merendino N. (2017). Impact of Omega-3 Fatty Acids on the Gut Microbiota. Int. J. Mol. Sci..

[129] Li J., Chen Y., Shi Q., Sun J., Zhang C., Liu L. (2023). Omega-3 polyunsaturated fatty acids ameliorate PM2.5 exposure induced lung injury in mice through remodeling the gut microbiota and modulating the lung metabolism. Environ. Sci. Pollut. Res. Int..

[130] Wang H., Wang Y. (2024). What Makes the Gut-Lung Axis Working? From the Perspective of Microbiota and Traditional Chinese Medicine. Can. J. Infect. Dis. Med. Microbiol..

[131] Xu L., Ho C.T., Liu Y., Wu Z., Zhang X. (2022). Potential Application of Tea Polyphenols to the Prevention of COVID-19 Infection: Based on the Gut-Lung Axis. Front. Nutr..

[132] Wang K., Hu S. (2023). The synergistic effects of polyphenols and intestinal microbiota on osteoporosis. Front. Immunol..

[133] Corrêa T.A.F., Rogero M.M., Hassimotto N.M.A., Lajolo F.M. (2019). The Two-Way Polyphenols-Microbiota Interactions and Their Effects on Obesity and Related Metabolic Diseases. Front. Nutr..

[134] Ali A., Wu L., Ali S.S. (2024). Vitamin D and the microbiota connection: Understanding its potential to improve COPD outcomes. Egypt. J. Bronchol..

[135] Albedewi H., Bindayel I., Albarrag A., Banjar H. (2022). Correlation of Gut Microbiota, Vitamin D Status, and Pulmonary Function Tests in Children with Cystic Fibrosis. Front. Nutr..

[136] Biazzo M., Deidda G. (2022). Fecal Microbiota Transplantation as New Therapeutic Avenue for Human Diseases. J. Clin. Med..

[137] Brandt L.J., Aroniadis O.C. (2013). An overview of fecal microbiota transplantation: Techniques, indications, and outcomes. Gastrointest. Endosc..

[138] Halaweish H.F., Boatman S., Staley C. (2022). Encapsulated Fecal Microbiota Transplantation: Development, Efficacy, and Clinical Application. Front. Cell. Infect. Microbiol..

[139] Gianotti R.J., Moss A.C. (2017). Fecal Microbiota Transplantation: From *Clostridium difficile* to Inflammatory Bowel Disease. Gastroenterol. Hepatol..

[140] Al K.F., Craven L.J., Gibbons S., Parvathy S.N., Wing A.C., Graf C., Parham K.A., Kerfoot S.M., Wilcox H., Burton J.P. (2022). Fecal microbiota transplantation is safe and tolerable in patients with multiple sclerosis: A pilot randomized controlled trial. Mult. Scler. J. Exp. Transl. Clin..

[141] Borody T., Campbell J., Torres M., Nowak A., Leis S. (2011). Reversal of Idiopathic Thrombocytopenic Purpura [ITP] with Fecal Microbiota Transplantation [FMT]: 941. Am. J. Gastroenterol..

[142] Laeeq T., Vongsavath T., Tun K.M., Hong A.S. (2023). The Potential Role of Fecal Microbiota Transplant in the Reversal or Stabilization of Multiple Sclerosis Symptoms: A Literature Review on Efficacy and Safety. Microorganisms.

[143] Jang Y.O., Lee S.H., Choi J.J., Kim D.H., Choi J.M., Kang M.J., Oh Y.M., Park Y.J., Shin Y., Lee S.W. (2020). Fecal microbial transplantation and a high fiber diet attenuates emphysema development by suppressing inflammation and apoptosis. Exp. Mol. Med..

[144] Tang Y., Chen L., Yang J., Zhang S., Jin J., Wei Y. (2024). Gut microbes improve prognosis of *Klebsiella pneumoniae* pulmonary infection through the lung-gut axis. Front. Cell. Infect. Microbiol..

[145] Clinical Trial ID: NCT05755308. Faecal Microbiota Transplantation to Ameliorate Nintedanib-Induced Diarrhea in Patients with Idiopathic Pulmonary Fibrosis (BIOFEV). NCT05755308.

[146] Perrot C.Y., Karampitsakos T., Herazo-Maya J.D. (2023). Monocytes and macrophages: Emerging mechanisms and novel therapeutic targets in pulmonary fibrosis. Am. J. Physiol. Cell Physiol..

[147] D’Souza W.N., Douangpanya J., Mu S., Jaeckel P., Zhang M., Maxwell J.R., Rottman J.B., Labitzke K., Willee A., Beckmann H. (2017). Differing roles for short chain fatty acids and GPR43 agonism in the regulation of intestinal barrier function and immune responses. PLoS ONE.

